# Ratiometric Two-Photon Near-Infrared Probe to Detect DPP IV in Human Plasma, Living Cells, Human Tissues, and Whole Organisms Using Zebrafish

**DOI:** 10.1021/acssensors.2c02025

**Published:** 2023-02-27

**Authors:** Javier Valverde-Pozo, Jose M. Paredes, Thomas J. Widmann, Carmen Griñan-Lison, Giada Ceccarelli, Antimo Gioiello, M. Eugenia Garcia-Rubiño, Juan A. Marchal, Jose M. Alvarez-Pez, Eva M. Talavera

**Affiliations:** † Nanoscopy-UGR Laboratory, Department of Physical Chemistry, Faculty of Pharmacy, Unidad de Excelencia en Quimica Aplicada a Biomedicina y Medioambiente (UEQ), University of Granada, C. U. Cartuja, 18071 Granada, Spain; ‡ GENYO, Centre for Genomics and Oncological Research, Pfizer/University of Granada/Andalusian Regional Government, 18016 Granada, Spain; § Instituto de Investigación Biosanitaria (ibs.GRANADA), 18012 Granada, Spain; ∥ UGC de Oncología Médica, Complejo Hospitalario de Jaen, 23007 Jaen, Spain; ⊥ Laboratory of Medicinal and Advanced Synthetic Chemistry (Lab MASC), Department of Pharmaceutical Sciences, University of Perugia, 06123 Perugia, Italy; # Centre for Biomedical Research (CIBM), Biopathology and Regenerative Medicine Institute (IBIMER), University of Granada, 18100 Granada, Spain; ∇ Department of Human Anatomy and Embryology, Faculty of Medicine, University of Granada, 18016 Granada, Spain

**Keywords:** DPP IV, ratiometric fluorescent sensor, NIR probe, two-photon excitation, bioimaging

## Abstract

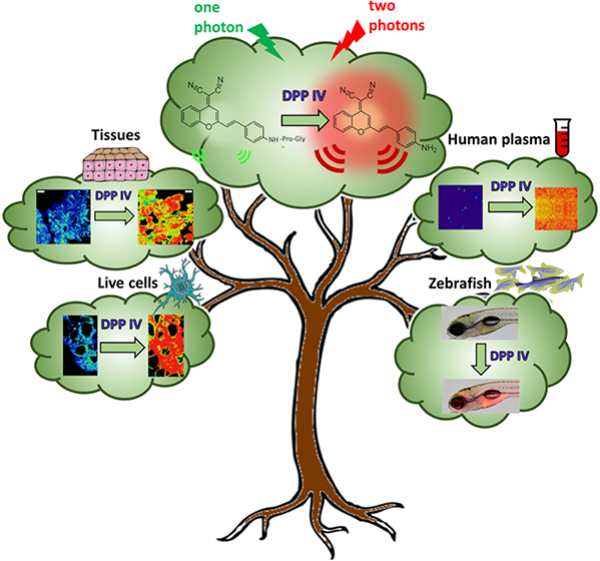

DPP IV, otherwise known as CD26 lymphocyte T surface antigen, is a transmembrane glycoprotein also found in circulation in the blood. It plays an important role in several processes like glucose metabolism and T-cell stimulation. Moreover, it is overexpressed in renal, colon, prostate, and thyroid human carcinoma tissues. It can also serve as a diagnostic in patients with lysosomal storage diseases. The biological and clinical importance of having readouts for the activity of this enzyme, in physiological and disease conditions, has led us to design a near-infrared (NIR) fluorimetric probe that also has the characteristics of being ratiometric and excitable by two simultaneous NIR photons. The probe consists of assembling an enzyme recognition group (Gly-Pro) (Mentlein, 1999; Klemann et al., 2016) on the two-photon (TP) fluorophore (derivative of dicyanomethylene-4*H*-pyran, DCM-NH_2_) disturbing its NIR characteristic internal charge transfer (ICT) emission spectrum. When the dipeptide group is released by the DPP IV-specific enzymatic action, the donor–acceptor DCM-NH_2_ is restored, forming a system that shows high ratiometric fluorescence output. With this new probe, we have been able to detect, quickly and efficiently, the enzymatic activity of DPP IV in living cells, human tissues, and whole organisms, using zebrafish. In addition, due to the possibility of being excited by two photons, we can avoid the autofluorescence and subsequent photobleaching that the raw plasma has when it is excited by visible light, achieving detection of the activity of DPP IV in that medium without interference.

The general principle of an enzyme activity assay is the use of specific enzyme substrates that interact with the enzyme, identifying the changes in substrate concentration by means of the appearance of some signal. Among the employed signals, fluorescence microscopy imaging has been considered a favorable method for detecting enzyme activity *in vivo* and is broadly used in preclinical research due to its ability to achieve real-time investigation of physiological and pathological processes with high sensitivity and high spatiotemporal resolution,^1−3^ making it possible to map enzymes in their native environment with high specificity.^4^ Therefore, microscopy imaging can help to detect unusual enzyme expression before changes in the morphology of unhealthy tissue occur and aid in the early diagnosis of diseases.

Fluorescence microscopy imaging requires appropriate probes that can be activated by specific enzymes to generate analytical signals.^5^ Therefore, the development of highly specific and high-resolution imaging probes is crucial for the precise detection of enzyme activity. In a large number of cases, enzymatic fluorescent probes requiring ultraviolet–visible (UV–vis) light excitation have been employed with one-photon microscopy (OPM). This practically excludes their use for investigating enzyme activities *in vivo* since it is difficult to obtain clear images of enzyme distribution in biological samples at a certain depth due to the absorption of the excitation light, the scattering properties of the tissues, and their autofluorescence, which all result in degradation of image resolution.^6^ Therefore, it is an essential requisite that both the incident and emitted light be of a long wavelength (red or near-infrared [NIR]) to achieve deep penetration inside the interior of a living organism, as well as lower fluorescence background from the organism, which allows a better three-dimensional localization of the probe.^7^

To avoid these drawbacks, a technique can be used that consists of the excitation of the fluorophore by means of two simultaneous photons of a wavelength that doubles or exceeds the wavelength needed to excite the same fluorophore with a single photon.^8,9^ Two-photon absorption was predicted by Nobel laureate Maria Goeppert Mayer in 1931^10^ and was applied by Webb in a cellular environment in 1990.^11^ Two-photon microscopy (TPM) uses NIR photons as an excitation source for fluorophores, resulting in deeper tissue images in biological systems. Moreover, TPM is suitable for three-dimensional resolution for *in vivo* enzyme activity studies, so TPM is gaining immense support for clinical optical imaging applications.^12^

New advances in microscopy instrumentation have made it possible to overcome the resolution limit, which has allowed fluorescence microscopy images to be brought to the nanometer scale. STED (stimulated emission depletion) microscopy with DCM-NH_2_ as a fluorophore was proposed in our laboratory to locate hot spots images of catalytic activity in Gram-negative bacteria that express peptidase N (pepN).^4^

DPP IV, also known as CD26 lymphocyte T surface antigen, was first reported as glycylproline naphthylamidase by Hopsu-Havu and Glenner.^13^ DPP IV is a transmembrane glycoprotein of 110 kDa MW expressed constitutively in a dimeric form (220 kDa) in a variety of cell types (prostate, kidney, liver, and epithelial cells). DPP IV is anchored in the plasma membrane with a type II orientation by means of a short cytoplasmic tail (amino acids 1–6), a transmembrane domain (TMD) (amino acids 7–28), a flexible region (residues 29–39), and a C-terminal extracellular domain (residues 40–766) containing catalytic activity.^14,15^ In addition to being membrane-bound, DPP IV is also found in circulation. Soluble DPP IV present in the plasma lacks the intracellular tail and transmembrane domain of the protein but retains substantial enzymatic activity.^16^ DPP IV shows enzymatic activity, being specific for a proline (Pro) residue at the penultimate position of the peptide chain, and hydrolyzes on the carboxyl side of this residue. The Pro residue can be substituted by alanine (Ala) or hydroxyproline (Hyp), although the rates of hydrolysis for these substrates are much lower than the rates of hydrolysis for the corresponding substrate containing Pro.^17,18^

DPP IV, involved in numerous pathological processes by regulating T-cell stimulation, plays an important role in several routes, such as glucose metabolism; therefore, DPP IV has been considered a target for the treatment of type 2 diabetes.^19^ Their inhibitors have been introduced to clinics as a class of oral hypoglycemic drugs (called gliptins) that are commonly used to treat type 2 diabetes mellitus and have been demonstrated to efficiently enhance endogenous insulin secretion.^20^ DPP IV has previously been associated with the start and progression of several human cancer types; hence, it is considered an important molecular marker and therapeutic target for cancer.^21,22^ DPP IV is overexpressed in human renal carcinoma tissues, and its blockage reduced several cancer-related processes in the human renal carcinoma cell line Caki-2.^23^ DPP IV has also been shown to be overexpressed in several human colon cancer tissues and in the human Caco-2 colorectal cancer cell line.^24,25^ In cancerous prostate, the DPP IV activity was enlarged 2-fold versus benign prostatic hyperplasia. Moreover, an elevation of its activity was also found in the peripheral zone of the prostate, where most prostate cancers arise.^26^ In humans, higher DPP IV levels in cancerous versus normal prostate tissue were correlated with prostate-specific antigen (PSA) level, cancer phase, and both tumor residue and size.^27^ In addition, DPP IV activity has been proposed as a marker for thyroid carcinomas^28^ and can also serve as a first-level diagnostic procedure to recognize patients with lysosomal storage diseases.^29^

Several methods for evaluating DPP IV activity have been established. Spectrophotometric activity assays can be performed by hydrolyzing the chromogenic substrate glycyl-prolyl-β-naphthylamide^13^ or through the measurement of the *p*-nitroaniline liberated from glycylproline *p*-nitroanilide.^30,31^ Fluorogenic substrates such as bis-(Ala-Pro)_2_-Rhod110^32^ or glycylprolylglycylprolyl-9-di-3-sulfonyl-propylaminobenza[*a*]phenoxazonium perchlorate,^33^ lanthanide metal ions for time-resolved fluorescence probes,^34^ or fluorescent probes with aggregation-induced emission (AIE) characteristics have been developed for DPP IV inhibitor screening due to the pharmacotherapeutic interest that inhibitors of DPP IV have in the treatment of type 2 diabetes.^35^ Therefore, a ratiometric two-photon (TP) fluorescent probe whose hydrolysis releases N-butyl-4-amino-1,8-naphthalimide and fluoresces with a maximum at 535 nm was developed by Zou et al.^36^ To monitor the *in vivo* DPP IV enzyme activity in biological systems in real time and with three-dimensional resolution, Guo and co-workers^37^ recently proposed an NIR fluorescent probe composed of a glycyl-prolyl peptide and a hemicyanine dye. However, neither of the fluorescent probes meet the three characteristics of being ratiometric, excitable by two photons and emitting NIR fluorescence.

Herein, a highly selective ratiometric two-photon NIR fluorescent probe that fluoresces with a maximum at 662 nm was designed, synthesized, and photophysically characterized. The new probe has been used for imaging DPP IV in human plasma, living Caco-2 colorectal cancer cells, tumor-bearing pancreatic tissue, and zebrafish embryos and larvae. The probe response is reached by the assembly of an enzyme-recognizing group (Gly-Pro) on the TP fluorophore (dicyanomethylene-4*H*-pyran derivative, DCM-NH_2_), forming an enzyme-sensitive donor–acceptor (D–A) system showing high ratiometric fluorescence output. Moreover, the possibility of nonlinear two-photon excitation along with ratiometric detection has been exploited to analyze DPP IV in raw plasma.

## Experimental Section

### Reagents and Standards

Dimethyl sulfoxide (DMSO), phosphate-buffered saline (PBS), 3-(4,5-dimethylthiazol-2-yl)-2,5-diphenyltetrazolium bromide (MTT) and the reagents and solvents used for DCM-NH-Pro-Gly synthesis were purchased from Sigma-Aldrich (St. Louis, MO). With the exception of the enzyme alanine aminopeptidase (ANEP), which was produced and purified as previously described,^4^ all other enzymes used, DPP IV, tyrosinase (TYR), acetylcholinesterase (AChE), lipase (PNLIP), dipeptidyl peptidase VIII (DPP VIII), fibroblast activation protein α (FAP), and leucine aminopeptidase (LAP) plus the enzyme inhibitor sitagliptin, were purchased commercially from Sigma-Aldrich. All of them were of the highest-quality grade.

### Sample Preparation

A 0.5 mM stock solution of DCM-NH-Pro-Gly dye was prepared in deuterated DMSO for purity testing by nuclear magnetic resonance. Unless otherwise indicated, the experimental samples were prepared in a 7/3 v/v mixture of PBS/DMSO buffer solution.

### Instrumentation

Steady-state fluorescence emission spectra and kinetics were obtained by a Jasco FP-8 300 spectrofluorometer (Jasco, Tokyo, Japan). UV–visible absorption spectrophotometry was carried out using a Cary 60 UV–visible spectrophotometer (Agilent, Santa Clara, CA). Both the fluorometer and the absorption spectrophotometer have a temperature controller.

Single-photon images were collected with a confocal microscope (Abberior Instruments GmbH, Heidelberg, Germany) supplied with a pulsed excitation laser (450 nm, 40 MHz) and a pulsed STED laser (775 nm, 40 MHz). The microscope has a UPlanSApo 1.4 NA, 100× objective oil immersion. The pinhole size was set to 1 Airy Unit. The collected fluorescence was separated by a 560LP dichroic directed to an avalanche-photodiode (APD) and hybrid photomultiplier tube (HPMT) detectors after passing through 685/75 and 545/25 filters, respectively.

Two-photon imaging was performed using a confocal MicroTime 200 fluorescence microscope system (PicoQuant GmbH, Berlin, Germany). The excitation source was a Chameleon Discovery NX tunable laser (Coherent Laser Group, Santa Clara, CA) used at an excitation wavelength of 800 nm. The repetition rate was modified by a pulse selector (APE Angewandte Physik & Elektronik GmbH, Berlin, Germany) using two acoustic-optic Bragg cells to reduce the frequency from 80 MHz to 40 MHz. The excitation beam passed through an achromatic quarter-wave filter (AQWP05-M-600, Thorlabs, Jessup, MD) and was directed by an F73–705SG dichroic mirror (AHF/Chroma, Tübingen, Germany) to an inverted microscope system (IX-71, Olympus, Tokyo, Japan) with an oil immersion objective (1.4 NA, 100×). Fluorescence emission was collected with a 550 nm longpass filter (AHF/Chroma, Germany) and directed to a 150 μm pinhole. The emission from the sample was split into two detection channels after passing through a 600 DCXR dichroic beam splitter (AHF/Chroma), and then through bandpass filters, 685/70 (Semrock/AHF) until one detector and through 520/35 filter (Semrock/AHF) to the other detector. The detectors used were two different single-photon avalanche diodes (SPADs) (SPCM-AQR 14, PerkinElmer, Waltham, MA).

Zebrafish embryos were imaged on a Nikon SMZ18 fluorescent stereo microscope with a color DS-Ri2 digital camera (16.25 megapixels) using filter settings for red fluorescent protein (RFP) and under a Zeiss LSM710 confocal microscope (Jena, Germany) with a 10x objective, recording brightfield and red and infrared fluorescent light (540 and 680 nm) after excitation with a 458 nm laser.

### Clog *P* Calculation and Image Processing

The Clog *P* values of both compounds were calculated using ChemDraw Professional v20 (PerkinElmer, Waltham, MA). Every image was exported as matrix data and analyzed using Fiji Is Just ImageJ.^38^ The analysis was performed taking two channels separately. Ratiometric values between the red and green channels were obtained by homemade macros described in the SI.

### Cell Line and Cell Culture

The human colon cancer Caco-2 cell line was obtained from American Type Culture Collection (ATCC, Manassas, VA). The cell line was cultured in Minimum Essential Medium Eagle (EMEM; Sigma-Aldrich, St. Louis, MO) supplemented with 10% heat-inactivated fetal bovine serum (FBS) (BioWhittaker; Lonza, Basel, Switzerland) and with 1% of a solution of penicillin/streptomycin (10 000 U mL^–1^ penicillin G and 10 mg mL^–1^ of streptomycin; Sigma-Aldrich, St. Louis, MO), and the solution was maintained at 37 °C in an atmosphere containing 5% CO_2_.

### Cytotoxicity Assay *In Vitro*

Caco-2 (colon cancer) and BxPC-3 (pancreatic cancer) cell lines were seeded in 96-well plates in a concentration of 3000 cells/well in EMEM and Roswell Park Memorial Institute (RPMI) medium. After 3 days, cells were treated with the DCM-NH-Pro-Gly (2.5, 5, and 10 μM involving a percentage of DMSO equal to 0.5, 1, and 2%, respectively), other wells were treated with DMSO, in the same percentages (0.5, 1 and 2%) being both incubated for 15, 30 and 60 min. Moreover, cells not treated were used as a control. After these times, all cells were incubated with MTT^39^ for 4 h and the absorbance was measured at 570 nm on a Microplate reader Synergy HT, BIO-TEK.

### Generation of Subcutaneous Xenograft Tumors

To establish subcutaneous xenograft tumors, an eight-week-old male NODSCID γ mouse (NOD. Cg-Prkdcscid Il2rgtm1 Wjl/SzJ, NSG) was used. All procedures were approved by the Institutional Animal Care and Use Committee at the University of Granada (ethical code: 03/07/2017/086). The mice were housed and maintained at 20–24 °C, 50% relative humidity (RH), and a 10:14 h light/dark cycle with food and water provided ad libitum. A human pancreatic cancer cell line, BxPC-3, was used to generate subcutaneous xenograft tumors, by injection of 1 × 10^6^ cells in 0.05 mL of Matrigel and 0.05 mL of Roswell Park Memorial Institute (RPMI) medium using 26-gauge needles. When the tumor reached 300 mm^3^, the mouse was euthanized by cervical dislocation. For analysis by TPM, tumors were excised, fixed in 4% paraformaldehyde (PFA), embedded in optimal cutting temperature (OTC) compound, and selected using a cryotome at a thickness of 10 mm for further analysis.

### *In Vivo* Imaging of DPP IV in Zebrafish

Zebrafish (*Danio rerio*) were kept in fish tanks with constant water flow at 28 °C, following maintenance and breeding recommendations from the zebrafish handbook (https://zfin.org/zf_info/zfbook/zfbk.html). Wild-type males and females were set up in a breeding tank. Egg laying occurred shortly after the onset of light in the morning on the following day. Embryos were raised in E3 embryo medium (5 mM NaCl, 0.17 mM KCl, 0.33 mM CaCl_2_, 0.33 mM MgSO_4_, 10^–5^% methylene blue) at 28 °C until the desired stage (1, 3, 5, and 7 days postfertilization (dpf)). Embryos of the desired stage were incubated in E3 medium with 5 μM DCM-NH-Pro-Gly (1:100 of stock solution at 500 μM in DMSO) for 2 h and then briefly washed with fresh E3 medium. Controls were incubated in E3 medium with DMSO (1:100). Subsequently, anesthetized (0.1 to 0.15 mg mL^–1^ MS222) live embryos were imaged under the stereo microscope. For confocal microscopy, embryos were mounted in low-melting agarose in E3 medium and subsequently imaged. Three to four tile scans of *z*-stacks (*z*-distance 5 μm) per zebrafish embryo were stitched together using Fiji Is Just ImageJ.^38^ Maximum projections of *z*-stacks (*z*-distance 5 μm) of fluorescent signals from zebrafish embryos are shown.

### Synthesis

The design strategy of the probe DCM-NH-Pro-Gly was to react the carboxyl group of the dipeptide Gly-Pro with the amine group of DCM-NH_2,_ perturbing the internal charge transfer (ICT) state that controls the spectral properties of DCM-NH_2_ and causing the shift of its absorption and emission spectra toward shorter wavelengths due to the effect of electron-withdrawing from the amide bond. Therefore, the catalytic action of DPP IV will restore the ICT in the DCM-NH_2_ molecule, providing remarkable ratiometric fluorescence between the peaks of the bands from DCM-NH_2_ and DCM-NH-Pro-Gly.

**Scheme 1:**
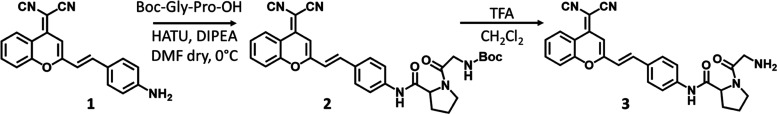
Synthesis of DCM-NH-Pro-Gly

DCM-NH_2_, (1), was first synthesized according to the literature.^40^ The reaction of (1) with N-Boc-Gly-Pro gave rise to the corresponding amide (2), which was modified into DCM-NH-Pro-Gly (3) after deprotection of the tert-butyloxycarbonyl (BOC)-protecting group with trifluoroacetic acid (TFA) (Scheme 1 and Figure S1 in the SI) (more details are available in the SI).

The synthetic method and the purification method are also detailed in the SI. The final product was characterized by ^1^H nuclear magnetic resonance (NMR), ^13^C NMR, and time-of-flight mass spectrometry (TOF MS), as shown in the SI.

### General Procedures for the DPP IV Activity Assay

Enzyme kinetic assays were carried out in PBS 100 mM (pH = 7.4)/DMSO, 7/3 v/v at 37 °C with shaking, adding the corresponding volumes from stock solutions to achieve the desired final concentration of enzyme and DCM-NH-Pro-Gly.

The enzyme inhibition study was performed using different concentrations of sitagliptin (0, 10, 100, and 250 μM) added to a PBS/DMSO (7/3, v/v), 100 mM, pH = 7.4 solution of 10 μM DCM-NH-Pro-Gly with 5 μg mL^–1^ DPP IV.

The DCM-NH-Pro-Gly enzyme selectivity assay was performed in PBS/DMSO 7/3 using different enzymes at a concentration of 5 μg mL^–1^.

In plasma, kinetic studies were carried out by adding only 15% DMSO. Plasma was collected from healthy volunteers and diabetic patients.

All cellular and *in vivo* studies were performed at a concentration of 5 μM DCM-NH-Pro-Gly. Enzyme inhibition studies in cells were carried out at a concentration of 5 μM DCM-NH-Pro-Gly and 50 μM sitagliptin.

## Results and Discussion

### Spectral Properties of the Probe and Its Response toward DPP IV

First, we performed spectral characterization of both the probe (DCM-NH-Pro-Gly) and the reaction product (DCM-NH_2_). Absorption spectra show maxima at 442 and 480 nm in PBS/DMSO (7/3, v/v) (see Figure S2 in the SI). The emission of both compounds was characterized by maxima at 550 nm (Φ = 0.10 ± 0.01%) and 662 nm (Φ = 0.63 ± 0.09%) under the same conditions.

The influence of the solvent on the photophysics of dicyanomethylene-4*H*-pyran (DCM) has been previously studied.^41^ Thus, the redshift of the emission maximum with increasing solvent polarity is caused by an intramolecular charge transfer and concomitant increase of the dipole moment upon excitation. Fluorescence lifetime studies of DCM in a large number of solvents further confirmed that the nature of the solvent plays an important role in the decay processes of the excited state;^42^ therefore, the solvent choice for the *in vitro* studies with DCM is of great importance.^43^ Based on the previous work and the results summarized in Figure S3, DMSO appears to be best suited in view of the longer fluorescence decay time and low photoisomerization efficiency.^42^ With a QY of Φ = 5.73 ± 0.09% for DCM-NH_2_ in DMSO, the presence of DMSO as a co-solvent is therefore well justified for the *in vitro* studies. Previous studies showed that in PBS buffer, the fluorescence signals decreased when less than 30% DMSO was used, further precipitating the dye shortly after dissolution.^4^

**Figure 1:**
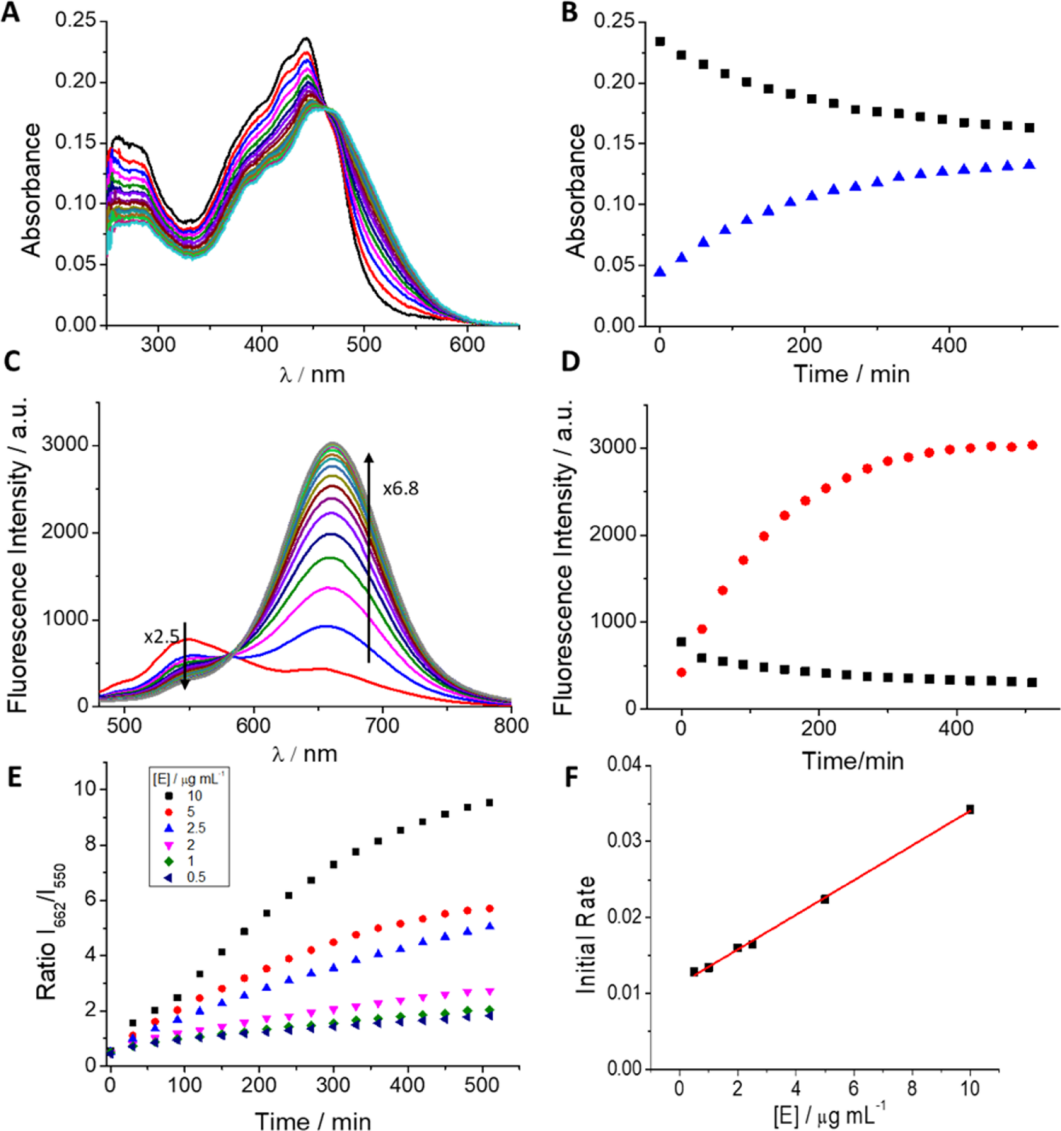
(A) Evolution of the absorption spectra of DCM-NH-Pro-Gly (10 μM) with DPP IV (10  μg mL^–1^) in PBS/DMSO (7/3, v/v) every 30 min for 8.5  h at 37  °C. (B) Maximum absorbance values at 440 nm (black line) and 500 nm (blue line) vs time. (C) Evolution of the emission spectra of DCM-NH-Pro-Gly (10 μM) with DPP IV (10 μg mL^–1^) observed every 30 min for 8.5 h by excitation at 463 nm at 37 °C. (D) Maximum fluorescence intensity at 662 nm (red line) and the intensity decrease at 550 nm (black line) vs time. (E) Ratiometric measurements of fluorescence signals of *I*_662_/*I*_550_ of DCM-NH-Pro-Gly (10 μM) with different enzyme concentrations vs time. (F) Initial rates from ratiometric measurements vs enzyme concentrations.

Next, we studied the response of the probe to the enzyme DPP IV. Figure 1 shows the absorption and emission spectra of PBS/DMSO (7/3, v/v), 100 mM, pH = 7.4 solutions of DCM-NH-Pro-Gly, every 30 min after DPP IV addition. As depicted in Figure 1A, the probe exhibited an absorption band with a sharp peak at ∼440 nm. Upon the addition of DPP IV, the absorption is red-shifted, forming a new band with a maximum at approximately 480 nm, characteristic of DCM-NH_2_,^4^ giving rise to an isosbestic point at 463 nm. Figure 1B shows the absorbance at the wavelengths of interest (440 and 480 nm). Figure S2A in the SI shows the absorptivity of both compounds. Our data indicate that at a very long reaction time, the absorbance of the reaction product (ε = 37 700 ± 1600 L mol^–1^ cm^–1^, see Figure S2A in the SI) should overcome the absorbance of the probe (ε = 17 600 ± 300 L mol^–1^ cm^–1^, see Figure S2A in the SI).

Concomitantly, the emission spectrum (λ_ex_ 463 nm, at the isosbestic point) of the probe shows a relatively low fluorescence band centered at 550 nm that, after the addition of DPP IV, gradually disappears over time and gives rise to another very intense emission band with a peak at 662 nm, as shown in Figure 1C. The decrease in the maximum of DCM-NH-Pro-Gly (λ_em_ = 550 nm) was approximately 2.5 times, while the increase at 662 nm achieved ∼6.8 times after 8.5 h of incubation. The emission spectra recorded after adding the enzyme are equal to the emission spectra shown from solutions of free DCM-NH_2_. Figure 1D shows the fluorescence intensity at the fluorescence maxima wavelengths. Notably, the ratio between the fluorescence signals at 662 and 550 nm, *I*_662_/*I*_550_, increased over time after the addition of DPP IV. To verify whether the ratio can be used as a measure to quantify enzymatic activity, we measured different kinetic curves every 30 min for 8.5 h using different DPP IV concentrations. Figure 1E represents the ratio values. This parameter is dependent on the DPP IV concentration, allowing its use to determine enzymatic activity.^35,44,45^

In addition, following Michaelis–Menten theory, the initial rates from the ratiometric measurements should depend on the enzyme concentration of the sample. We confirm that the initial rates of the ratiometric measurements depend on the DPP IV concentration. Figure 1F shows the graphical representations of the initial rates and the enzyme concentration showing an excellent linear relationship (*R*^2^ = 0.998) in the range of concentrations measured (0.5 and 10 μg mL^–1^).

To confirm that the enzymatic reaction releases DCM-NH_2_, we first performed an MS of the dye (see Figure S4). After identifying the peak M^+^, we incubated DCM-NH-Pro-Gly with DPP IV and monitored the increase of the M^+^ peak at different time points by an HPLC-MS (Figure S5) showing an increase over time. Finally, we made a calibration curve using different DCM-NH_2_ concentrations (see Figure S6A) and we calculated the DCM-NH_2_ released at different times (see Figure S6B). Our data reflect a plateau at approximately 300 min, a similar value as obtained by absorption and emission spectroscopy. Additionally, we also determined whether the increase in fluorescence was due to the action of DPP IV performing inhibition studies using sitagliptin, a selective inhibitor of DPP IV^46,47^ (see Figure S7 in the SI). Figure S8 in the SI depicts the *I*_662_/*I*_550_ ratios. As observed, the incorporation of sitagliptin slows the reaction rate and produces a decrease in the 662 nm peak, showing that sitagliptin inhibits the action of the enzyme. Therefore, since sitagliptin scarcely affects the emission of DCM-NH_2_, the substantial NIR fluorescent band with a maximum at 662 nm must be attributed to DCM-NH_2_ released by specific cleavage of the amide bond due to DPP IV activity. Moreover, we checked the efficiency of the enzyme activity at different pH values and temperatures (Figure S9), showing good catalytic activity at pH 7.5 and 37 °C.

### Selectivity of DCM-NH-Pro-Gly

**Figure 2:**
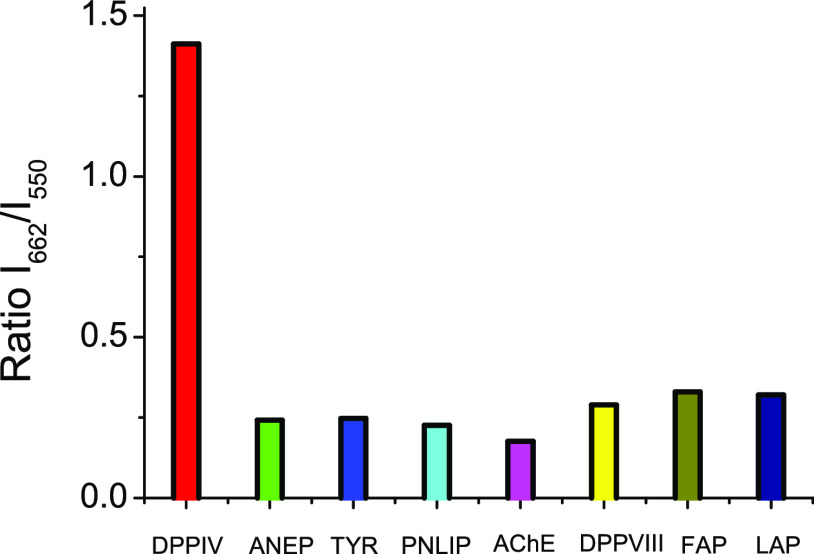
Ratiometric measurements of fluorescence signals of *I*_662_/*I*_550_ of DCM-NH-Pro-Gly (10 μM) in PBS/DMSO (7/3, v/v) after 80 min of incubation in the presence of different enzymes at the same concentration (5 μg mL^–1^) by excitation at 463 nm at 37 °C.

To explore the applicability of DCM-NH-Pro-Gly for sensing DPP IV in biological samples, the specificity of the sensor was investigated. In Figure 2, the fluorescence *I*_662_/*I*_550_ ratio enhancement in the presence of DPP IV is compared to increases in the fluorescence *I*_662_/*I*_550_ ratio calculated when the samples were incubated with other related enzymes, such as ANEP, TYR, AChE, PNLIP, DPP VIII, FAP, and LAP. The evident augmentation of the *I*_662_/*I*_550_ ratio in the samples incubated in the presence of DPP IV versus the same fluorescence ratio from the samples incubated with the other enzymes unambiguously demonstrates the high specificity of our probe toward the enzymatic action of DPP IV.

### Enzymatic Kinetics Parameters of DPP IV-Mediated DCM-NH-Pro-Gly Hydrolysis According to the Michaelis–Menten Model

To avoid tedious corrections, the solutions used in the calculation of the characteristic parameters of the reaction kinetics were excited at 550 nm wavelength, which practically excites only the reaction product, generating fluorescence signals proportional to the concentration of the reaction product DCM-NH_2_. We elaborate a Kavanagh law (see Figure S10 in the SI) to determine the DCM-NH_2_ concentration released in the course of the reaction.

To resolve the enzyme kinetics following the Michaelis–Menten model, after transforming the fluorescence intensity into reaction product concentration, the initial rates of the enzymatic reaction at different initial substrate concentrations were calculated from the recorded fluorescence signal from DCM-NH_2_ (Figures S11 and S12 in the SI). The Michaelis–Menten model is represented in Figure S13 in the SI. Linear regression fitting based on the Lineweaver–Burk equation provided the following values: *K*_M_ = 486 ± 46 μM and v_max_ = 0.588 ± 0.044 μM min^–1^ mg^–1^ (Figure S14 in the SI). As a result, the k_catalytic_ that we obtained is k_cat_ = 5.9 × 10^–4^ ± 0.2 × 10^–4^ μM min^–1^ μg^–1^ mL.

### Enzymatic Kinetics of DPP IV-Mediated DCM-NH-Pro-Gly Hydrolysis in Human Plasma

Due to the great importance of DPP IV as a potential biomarker in the diagnosis and treatment of tumors, type 2 diabetes mellitus, and other serious diseases, as well as in the development of hypoglycemic drugs, it is very interesting to investigate the applicability of DCM-NH-Pro-Gly for the quantitative and precise determination of DPP IV activity in human plasma.

To test the activity of DPP IV in plasma with our probe, we collected samples from healthy individuals and those with diabetes mellitus. Due to the native fluorescence of the plasma from the endogenous fluorophores that it has in solution, it is necessary to perform a study of both excitation and emission spectra to find the most favorable conditions for the analysis of the activity of DPP IV in raw plasma.

The excitation spectrum of plasma, in the visible region, with detection at 580 nm (in which the probe and the product of enzymatic reaction show an isoemissive point), consists of three bands with maxima at approximately 350, 440, and 510 nm (Figure S15 in the SI). The wavelength of 480 nm, which is where the DCM-NH_2_ probe has the absorption maximum, is located between two excitation peaks of the plasma so that in the activity analyses performed, the sample has always been excited at 480 nm to obtain maximum fluorescence intensity.

In Figure S16, the emission spectrum from the plasma under the experimental conditions used in our experiments (λ_ex_ = 480 nm) is represented, showing an emission peak ca. 550 nm, in concordance with the average native fluorescence spectrum of blood plasma from normal human subjects. Usually, human plasma shows two emission bands in the visible spectrum, one of them with a maximum at approximately 460 nm (attributed to fluorophores such as riboflavinoproteins, vitamin A, bilirubin, and lipoproteins) and another in the spectral range between 550 and 650 nm, which can be attributed to the presence of endogenous porphyrins,^48^ such as hematoporphyrin and protoporphyrin, which fluoresce in that wavelength range depending on the solvent polarity.^49^

On the other hand, the presence of a certain percentage of DMSO enhances the fluorescence of DCM-NH_2_.^4^ Therefore, we added different amounts of DMSO to a solution containing the products of the enzymatic reaction between DPP IV and our probe in plasma after reacting for 48 h at 37 °C. Figure S17 shows that the maximum emission was obtained when 15 or 20% DMSO was added, while the addition of 30% resulted in loss of the fluorescent signal and precipitation of some plasma components. To reduce the possible denaturation of DPP IV and achieve the maximum possible sensitivity, we used a percentage of DMSO equal to 15% in all our following experiments.

In addition to these spectral features, a strong photobleaching effect on porphyrins at light power densities that were not too high has been reported,^50^ such as we have observed in experiments in which plasma spectra have been collected at the same times as in experiments with DPP IV and the probe. Figure S18 represents the spectra showing the considerable effect of 50% photobleaching in the fluorescence band ca. 550 nm of the plasma after 24 h by collecting spectra every hour. Therefore, in experiments on the kinetics of the enzymatic reaction, the samples from healthy subjects were divided into two aliquots. DPP IV and DMSO up to 15% were added to one of the probes. The other aliquot was composed only of plasma at the same dilution and 15% DMSO. Emission spectra (excited at 480 nm) from the two aliquots were recorded every hour for 24 h. In Figure S19, raw spectra from the aliquot with probe and DPP IV are represented, and in Figure S20, the spectra corresponding to plasma/DMSO were subtracted, at each time, from those collected from the solution of DCM-NH-Pro-Gly 10 μM and DPP IV 10 μg mL^–1^ in plasma/DMSO. As seen in Figure S20, the calculation performed shows a fluorescence band with a maximum at approximately 635 nm with a shape similar to the shape of DCM-NH_2_ but slightly blue-shifted, probably due to the lower polarity of the medium.

A similar set of experiments was carried out with plasma from healthy subjects without adding external enzymes and from diabetic patients. The crude spectra collected corresponding to both samples of blood plasma with the probe are depicted in Figures S21 and S22, respectively. In both sets of experiments, those carried out with plasma from healthy subjects and plasma from diabetic patients, a new emission band appears with a maximum at approximately 635 nm that belongs to the product of the reaction with the probe that acts as a substrate of the enzymatic reaction with DPP IV. Importantly, in Figure S23, we compare the *I*_635_/*I*_540_ ratio derived from the spectra measured under the same conditions from healthy subjects and diabetic patients. Our results show a higher increase in intensity at 635 nm due to the higher presence of DPP IV in the diabetic plasma. Significantly, the slight increase in fluorescence intensity at 635 nm in the plasma from healthy subjects demonstrates that our probe is a useful tool as a sensor for the analysis of trace amounts of DPP IV in human plasma.

From the above discussion, it follows that the main drawback to measure the DPP IV activity in plasma by means of steady-state fluorescence is the strong plasma autofluorescence that appears at approximately 540 nm and its photobleaching with irradiation time (see Figure S18), making it impossible to perform reliable ratiometric measurements between the fluorescence at 635 and 540 nm.

To remove the autofluorescence coming from plasma, we considered using two-photon excitation to minimize that fluorescence. To check this idea, first, we checked the optimal excitation wavelength to achieve the maximum signal at each channel. For this, we recorded the fluorescent images of DCM-NH_2_ and DCM-NH-Pro-Gly at different excitation wavelengths from 720 to 1000 nm. We obtained the highest signal from both compounds at 800 nm (Figure S24).

**Figure 3:**
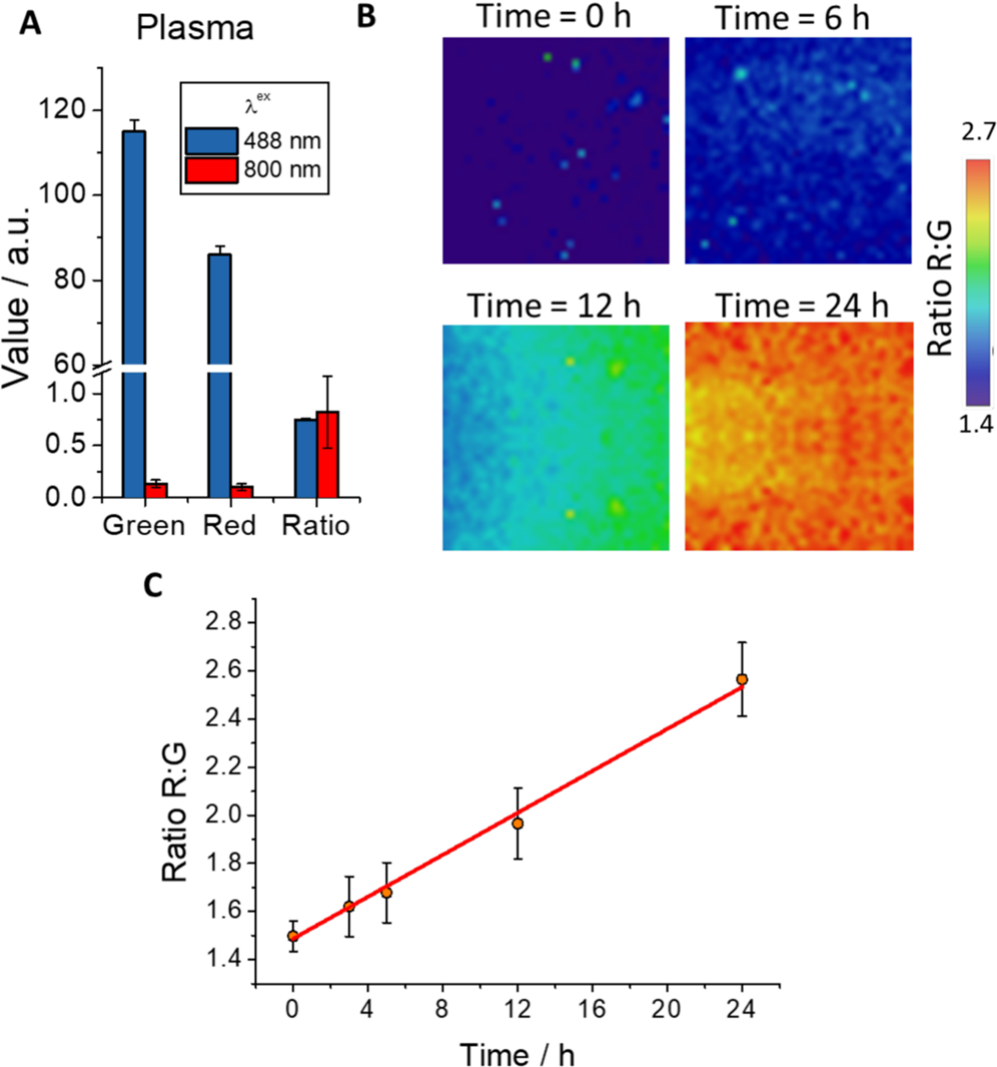
(A) Intensity values of green (λ_em_ = 533–557 nm) and red (λ_em_ = 648–722 nm) channels and ratio maps using one-photon excitation at 488 and two-photon excitation at 800 nm of blood plasma sample. Whiskers represent the standard error (SE). (B) Representative ratio R/G maps of healthy human blood plasma with DCM-NH-Pro-Gly with DPP IV (10 μg mL^–1^) at different incubation times (λ_ex_ = 800 nm). C) Ratio R/G average values of blood plasma with the probe DCM-NH-Pro-Gly images registered at different incubation times (λ_ex_ = 800 nm). Line represents a visual aid. Whiskers represent the SE.

Therefore, we performed experiments to measure the intensities in plasma using both detection channels (green and red) under the aforementioned experimental conditions. In Figure 3A, the intensities measured in both channels are represented using excitation wavelengths of 488 and 800 nm. As can be clearly observed, the use of two-photon excitation remarkably reduces the plasma autofluorescence in both detection channels, where the fluorescence measured when 800 nm was used as the excitation wavelength is negligible, probably due to a very low two-photon cross section from the fluorescent components of the plasma.

Once it was shown that excitation at 800 nm practically eliminated plasma autofluorescence, DCM-NH_2_ dye was added to another plasma sample. For microscopy experiments, we collected the fluorescence emission in two different channels (red channel, for the emission of the released product of the reaction, DCM-NH_2_, and the green channel for the emission coming mainly from DCM-NH-Pro-Gly). Thus, the ratio values were measured through the quotient of both channels (ratio R/G). Figure S25 shows the recovered fluorescence signals in both channels along with the ratio R/G, which corresponds to the unperturbed emission of the compound. We also performed experiments in which the DCM-NH-Pro-Gly probe was added to the plasma (Figure S26), obtaining the expected ratio corresponding to the unreacted probe, i.e., lower red fluorescence intensity, higher green fluorescence intensity and lower R/G ratio than DCM-NH_2_.

Finally, we carried out experiments in which the probe and the enzyme were added to the plasma, allowing them to react for 24 h and taking samples at various times for their measurement. Some representative ratio R/G images are depicted in Figure 3B. In this figure, it is possible to visualize color differences in the images at different incubation times, where the color change is caused by the cleavage of DCM-NH-Pro-Gly into DCM-NH_2_. The results of measuring different samples are represented in Figure 3C, which shows that the R/G ratio increases gradually over time. The rate of appearance of the products of the enzymatic reaction between DPP IV and a certain substrate has been used to calculate the activity of DPP IV in plasma from patients with diabetes.^36,51^

The effectiveness of the methodology developed with the DCM-NH-Pro-Gly probe opens a door to its future use in the *in situ* detection of DPP IV, allowing the diagnosis of diseases in which the enzyme is overexpressed in the blood. Of course, the proposed methodology must be optimized to provide quantification of enzyme activity. Currently, the necessary experiments for this purpose are being designed in our laboratories and will be published elsewhere.

### Fluorescence Imaging of DPP IV in Living Cells

One of the main advantages of optical probes is the possibility of measuring them in real time, *in vivo*, and *in situ*. These characteristics make them very attractive for use in biological samples. To prove the efficiency of DCM-NH-Pro-Gly as a DPP IV intracellular sensor, we selected the Caco-2 cell line as a cellular culture of interest. Caco-2 cells are from human colorectal adenocarcinoma, a disease reported to have higher DPP IV activity.

**Figure 4:**
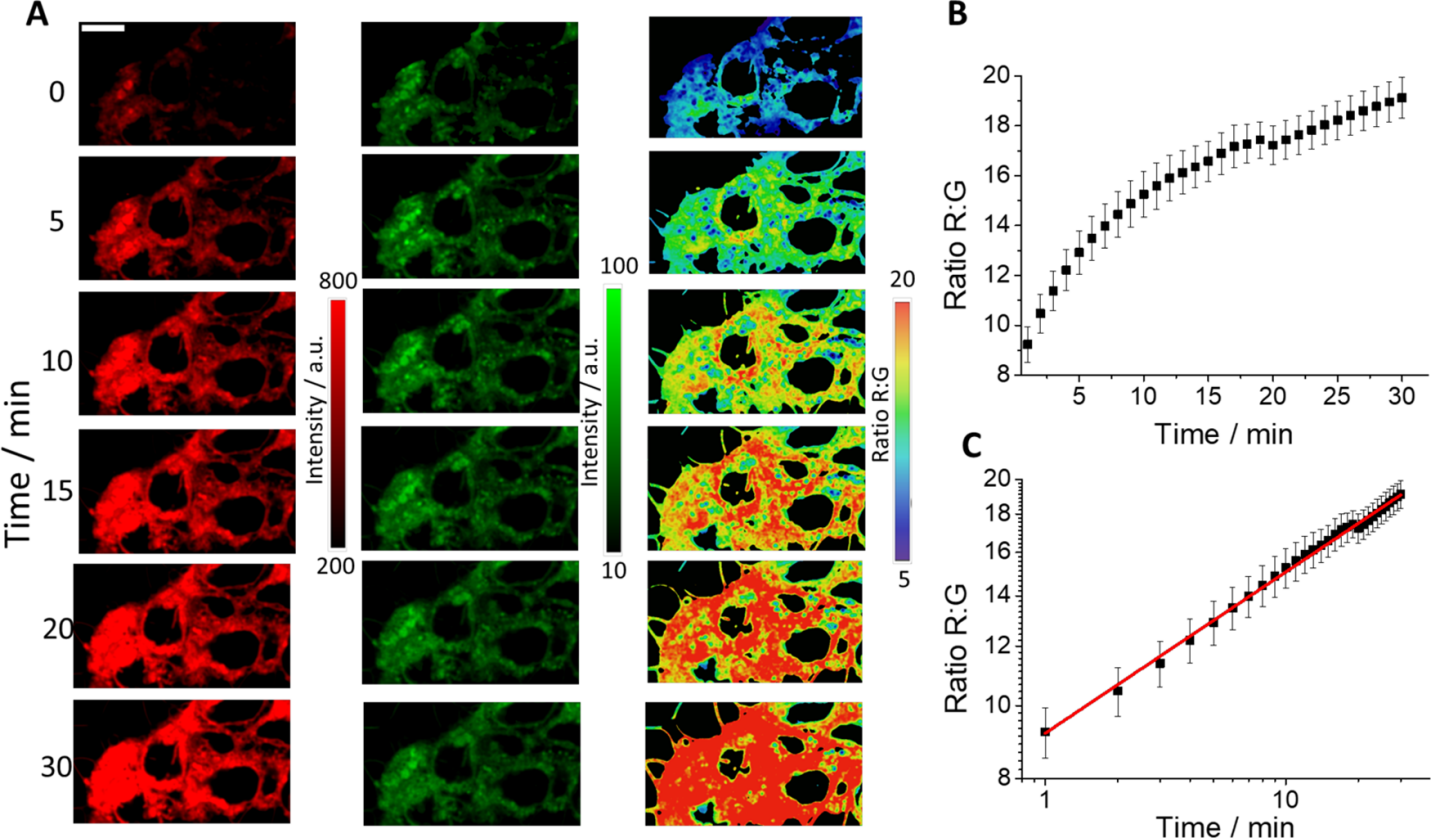
(A) Images of a representative sample of the live Caco-2 cell line incubated with DCM-NH-Pro-Gly (2.5 μM) recorded in the red (left, λ_ex_ = 450 nm, λ_em_ = 648–722 nm) and green (middle, λ_ex_ = 450 nm, λ_em_ = 533–557 nm) channels at six different time points. Ratio R/G images (right) obtained at the same times. (B) Representation of the average values of the ratio R/G from five independent experiments. Error bars represent SE. (C) Double-logarithmic representation of the kinetics. Error bars represent SE. The red line is a linear fit with an intercept of 0.964 ± 0.004 and a slope of 0.215 ± 0.003 (*R*^2^ = 0.995).

After the addition of DCM-NH-Pro-Gly to extracellular media, the probe penetrates inside the cells rapidly and spontaneously, as do similar chemical probes described in the literature.^4,52,53^ As soon as we added the probe, we observed an increase in the emission recorded in the red channel (Figure 4A), whereas the green channel registered a very slight decrease. Despite the relatively low QY of the dye, the acquired images show a good signal-to-noise ratio, which could be due to the large environment dependence of the quantum yield. Similar to DMSO, which yielded an order of magnitude increased QY relative to PBS/DMSO 7/3 (v/v) buffer, the lower polarity environment inside cells might also result in a significantly higher QY and an overall increased brightness. Consequently, the ratio R/G images showed an evident increase (Figure 4A). In Figure 4B, we represent the average ratio R/G values with respect to time. As expected, the kinetics showed a good intracellular growth pattern. The representation of these data on a double-logarithmic scale (Figure 4C) fits remarkably well with a linear fit, as evidence indicates that the R/G ratio arises only from probe cleavage and not from slow entry of the probe into the cells, confirming our hypothesis of fast intracellular penetration of the probe. The good cell membrane permeability can also be confirmed with the calculation of the Clog *P* values (the coefficient between *n*-octanol and water, a well-established measure of the hydrophilicity of the compound). Our obtained values were 2.615 and 2.877 for DCM-NH-Pro-Gly and DCM-NH_2,_ respectively. Our data are inside the range of the Lipinski rule for substances with good cell permeability (−0.5 < log *P* < 5).^54^

We compared the proliferation rate in two cell lines (Caco-2, and BxPC-3) after treatment with different concentrations of DCM-NH-Pro-Gly and DMSO at several times (see Figure S27). From the results obtained, we conclude that the DCM-NH-Pro-Gly compound is not toxic in Caco-2 cells, while for BxPC-3 cells the possible minimal toxicity is due to the DMSO in which DCM-NH-Pro-Gly is dissolved, and not to the chemical structure of this sensor itself.

**Figure 5:**
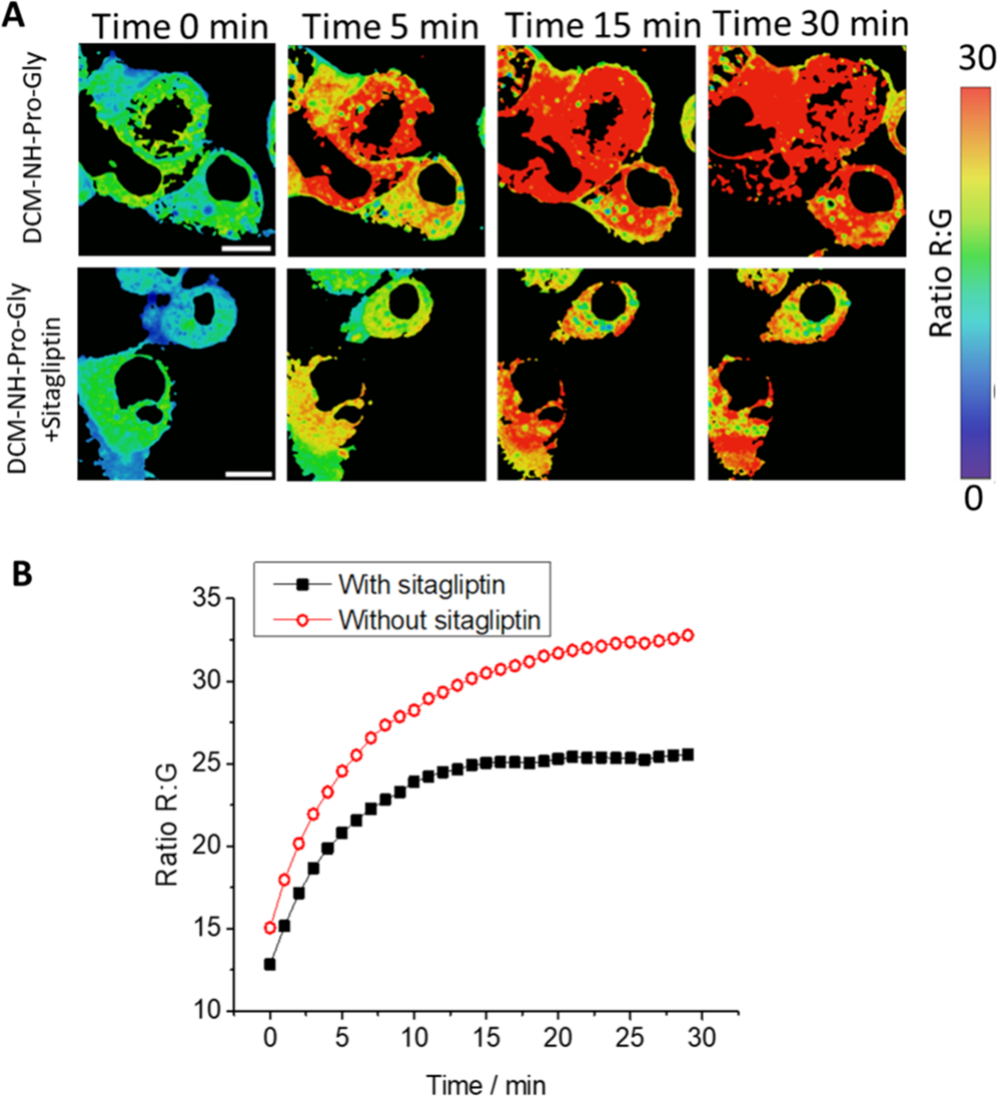
(A) R/G ratio maps of the live Caco-2 cell line at different times after adding DCM-NH-Pro-Gly (5 μM) without (top) and with (bottom) the DPP IV inhibitor sitagliptin (50 μM). λ_ex_ = 450 nm. Ratio image is calculated by dividing red (λ_em_ = 648–722 nm) and green (λ_em_ = 533–557 nm) channels. (B) Representation of the R/G ratios from microscopy images without (circles) and with (squares) sitagliptin. Scale bars represent 10 μm.

Finally, to confirm that DPP IV activity is responsible for the generation of red fluorescence, we measured the kinetics of the R/G ratio over 30 min in live Caco-2 cells in the presence and absence of sitagliptin. Figure 5A shows the ratio R/G images of a representative sample. A lower transition to high values is observable in the color scale selected in the cell culture with sitagliptin. Figure 5B represents the increase in the R/G ratio with respect to time. The addition of the inhibitor produces a slower rate reaction, as can be observed by the initial slope of both curves, and the production of a lower quantity of the product of the reaction, DCM-NH_2_, was observable in the final plateau value achieved in both cases.

**Figure 6:**
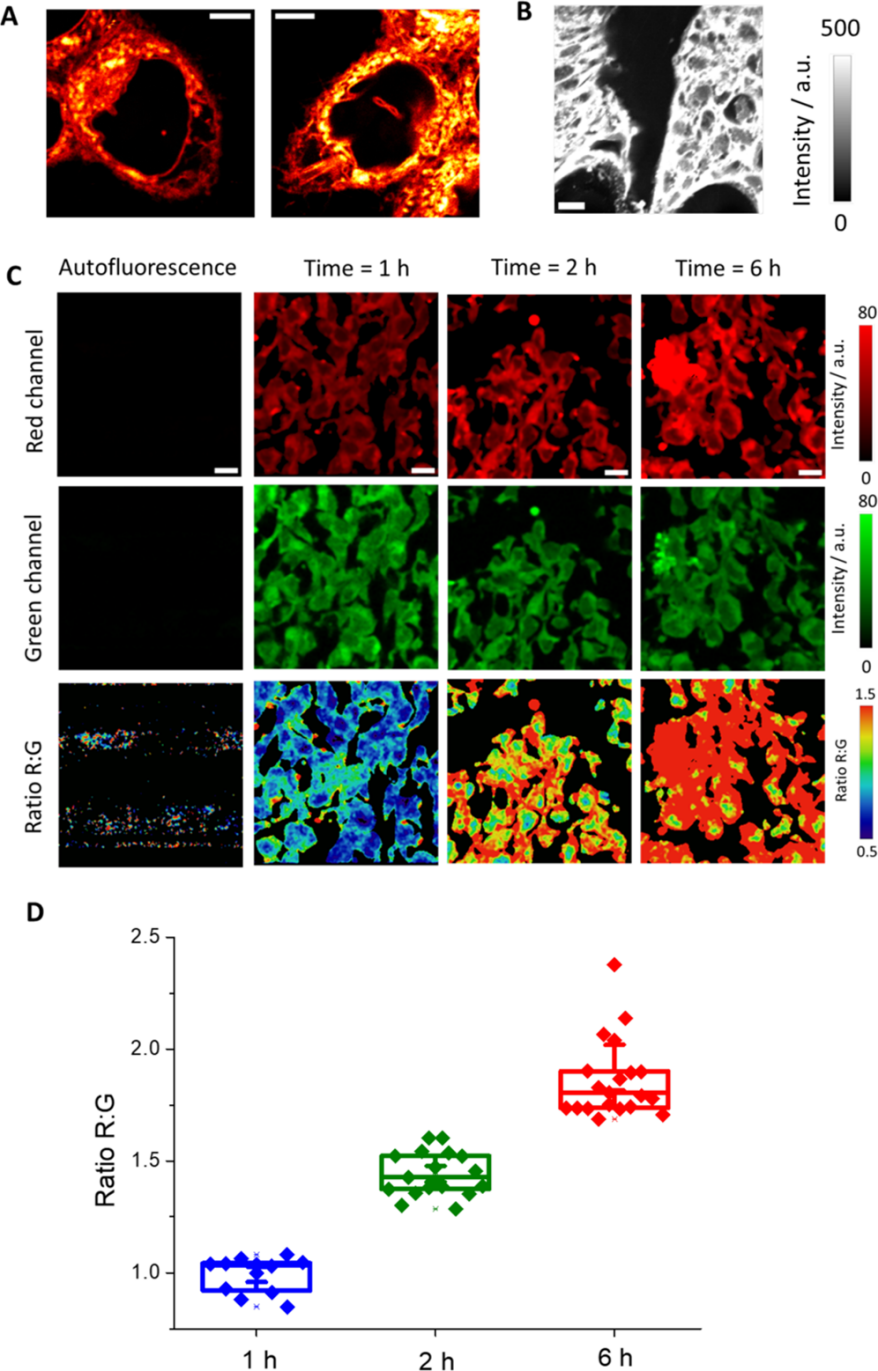
(A) Images obtained from live Caco-2 cells with two-photon excitation at 800 nm (λ_em_ = 650–720 nm). Scale bars represent 5 μm. (B) Representative image of the red channel obtained from BxPC-3 tumors using two-photon microscopy with excitation at 800 nm (λ_em_ = 650–720 nm). Scale bars are 10 μm. (C) Representative images of the intensity red (λ_em_ = 650–720 nm) and green (λ_em_ = 502–538 nm) channels and the ratio R/G images of BxPC-3 tumors after adding DCM-NH-Pro-Gly (10 μM) using two-photon microscopy with excitation at 800 nm. Scale bars are 10 μm. (D) Representation of the R/G ratio values from microscopy images. Boxes represent the 25th, 50th, and 75th percentiles. Whiskers represent the SE.

DCM-NH_2_ was reported to be usable in superresolution imaging and using two-photon excitation in bacterial bodies.^4^ With respect to superresolution, we checked the ability to use them in eukaryotic cells. The good resolution achieved with confocal microscopy due to the excellent fluorescent response of the probe makes the improvement in resolution accomplished with superresolution microscopy only slightly higher (see Figure S28 in the SI). On the other hand, we confirmed its ability to use in TPM by measuring live Caco-2 cells at an excitation wavelength of 800 nm. Figure 6A shows representative two-photon excitation images obtained. All of the images obtained by the various fluorescence microscopy techniques used show an intracellular accumulation in organelles of DCM-NH_2_, as described in the literature.^55^

Therefore, our imaging experiments with cell cultures, confirm the applicability of this probe as an intracellular sensor of DPP IV activity. These findings, combining the ability for two-photon excitation and the NIR emission of DCM-NH_2_, are promising characteristics to go further in biological applications.

### Imaging of DPP IV in Human Tumor-Bearing Pancreas Tissues

The imaging applied in tissues can provide important utilities in multiple aspects, including medical applications such as diagnosis or surgery. Taking advantage of this probe, we examined the ability of DCM-NH-Pro-Gly to detect DPP IV activity in tissues. For this purpose, we selected BxPC-3 tumors to test whether we could detect the NIR emission of DCM-NH_2_. We incubated the tissues in PBS with a solution of DCM-NH_2_ for 24 h. After this period, we washed with PBS and measured the emission intensity with two-photon excitation at 800 nm. Our data show a strong time-dependent increase of the average fluorescence intensity compared to control samples, which showed an almost negligible NIR emission (see Figure 6B,C).

Once we checked that we were able to recover the emission of DCM-NH_2_, we next incubated the tissues with the DCM-NH-Pro-Gly probe, and we measured the green and NIR emission just after 1, 2, and 6 h of incubation.

We observed again a negligible autofluorescence, whereas the probe at the initial time (1 h) showed a green and NIR emission with a similar intensity. However, after 2 h of incubation, we measured an increase in the NIR emission, whereas the green channel keeps with similar fluorescence. This behavior continues after 6 h of incubation. This increase should correspond with the cleavage of the probe DCM-NH-Pro-Gly due to the presence of DPP IV activity in the cell of the tissue releasing the compound DCM-NH_2_ (Figure 6C).

Finally, we represented the red-to-green ratio images at different incubation times with DCM-NH-Pro-Gly. As shown in Figure 6C, and as expected, the images showed a change in the ratio. Figure 6D shows the changes in the ratio values at these three incubation times. In a period of 6 h, we measured an increase in the ratio from ∼1.0 to ∼1.8.

Therefore, our results confirm the successful use of DCM-NH-Pro-Gly to detect DPP IV activity in *ex vivo* tissues.

### *In Vivo* Imaging of DPP IV in Zebrafish

Recently, DPP IV activity was found in zebrafish embryos and larvae; however, in this study, DPP IV activity was determined only qualitatively.^36,51^ In this work, we have gone farther, and we have completed, as far we know, for the first time, a quantitative study of the differences in DPP IV activity during 1, 3, 5, and 7 dpf in zebrafish embryos and larvae.

**Figure 7:**
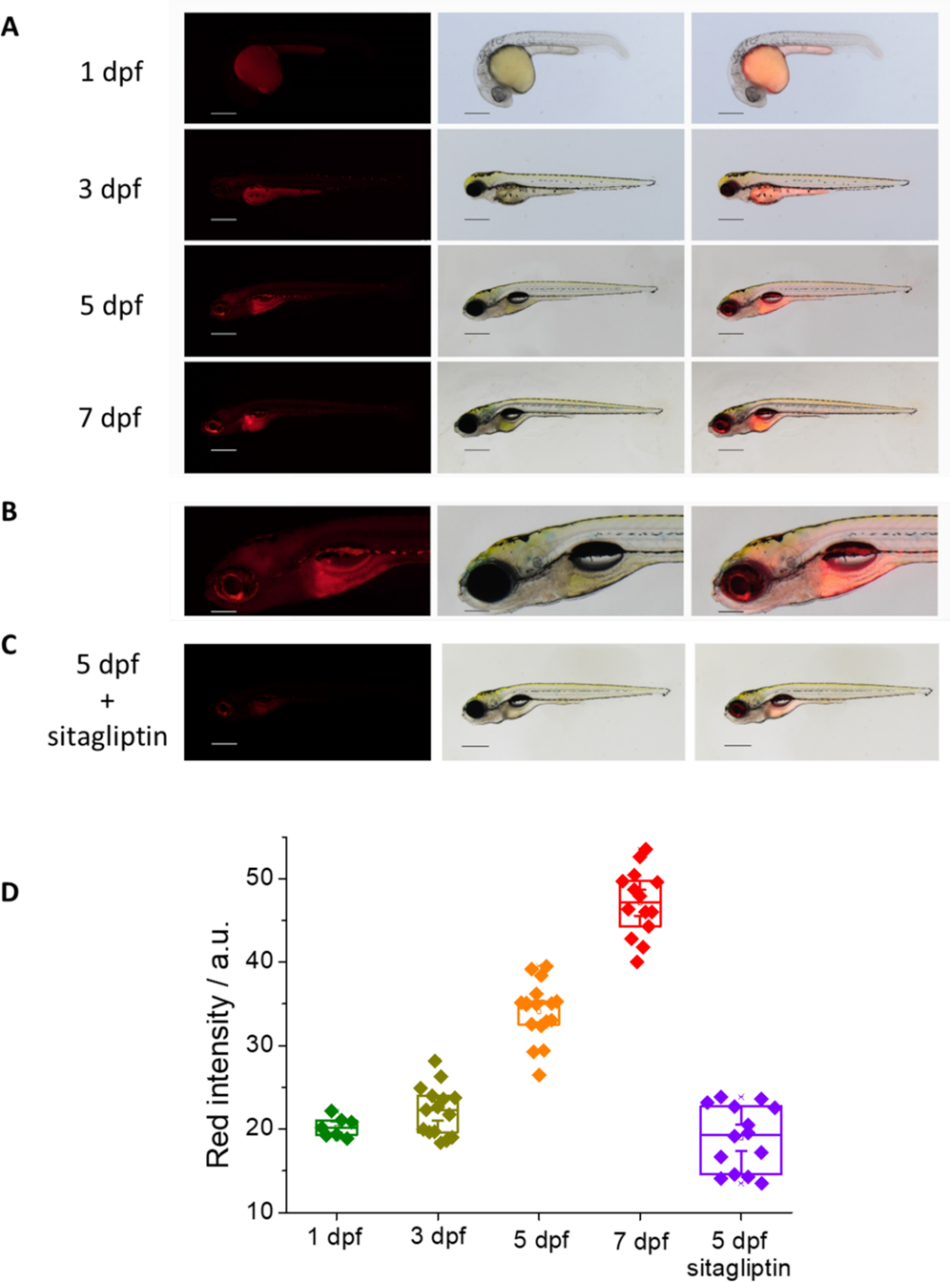
(A) Living zebrafish embryos and larvae incubated with 5 μM DCM-NH-Pro-Gly for 2 h at different dpf; red fluorescent (left), brightfield (center), and merge (right) images are shown measured by the stereo microscope (λ_ex_ = 458 nm, λ_em_ = 680 nm). Scale bars: 1 dpf: 250 μm, 3–7 dpf: 500 μm. (B) Detail (head with central nervous system) of a living zebrafish larva at 5 dpf. Scale bars: 200 μm. (C) Living zebrafish larva at 5 dpf preincubated for 3 h with 250 μM sitagliptin and incubated with 5 μM DCM-NH-Pro-Gly for 2 h. (D) Intensity values of NIR emission of zebrafish at different dpf, incubated with 5 μM DCM-NH-Pro-Gly in the presence or absence of the inhibitor sitagliptin. Boxes represent the 25th, 50th, and 75th percentiles. Whiskers represent the SE.

While zebrafish embryos and larvae control incubated with DMSO showed very little red autofluorescence in the yolk sac and eye (Figure S29 in the SI), all zebrafish stages incubated with the probe metabolized the original green substrate to a strong red and far red fluorescent derivative in both yolk sac and embryonic tissues (Figure 7). Zebrafish embryos at 1 dpf showed red fluorescence mainly in the yolk sac (Figure 7A, upper line), indicating high activity of DPP IV and/or high permeability of the yolk membrane for the probe. Zebrafish larvae at 3 dpf showed red fluorescence in the yolk sac and start to display an accumulation of red fluorescent metabolites in other larval tissues (Figure 7A, second line). In the older developmental stage, we measured (5 dpf, 7 dpf), the more red fluorescent derivative was generated in the fish (Figure 7A, third and fourth lines), indicating an increase in the levels of DPP IV activity with developmental stage. The most affected larval tissues are the central nervous system (CNS), especially the midbrain, hindbrain and spinal cord, eye, and inner organs, such as the throat and digestive tract. In contrast, skin and muscles did not show strong red fluorescent signals (Figure 7A,B). DPP IV is therefore predicted to be active in the yolk sac from zebrafish embryonic stages onward, while in larval stages, the enzyme is possibly active in the yolk sac, inner organs, and the CNS. The same pattern of red metabolite accumulation was also observed by confocal microscopy measurements (see Figures S30 and S31).

Additionally, a suitable control was performed with sitagliptin, as a DPP IV inhibitor, to be certain that the red fluorescence is due to the product generated after the enzyme action. For this purpose, we used a 5 dpf zebrafish that was preincubated with the inhibitor and then incubated with the same substrate concentration and for the same time. Indeed, the images obtained with the 5 dpf zebrafish that was not inhibited prior to the addition of the substrate display a strongly reduced red fluorescence (Figure 7C).

In addition to qualitatively describing the accumulation of red DCM-NH_2_ released in the different zebrafish tissues, we quantified the intensity coming from the NIR emission of the compound from the images recovered with the stereo microscope at different dpf. Our data showed a similar activity (with negligible differences) at 1 and 3 dpf, though a slight increment of the red emission in the 3 dpf with respect to the 1 dpf can be appreciated, which could indicate a small increase of DPP IV activity with respect to the previous stage.

However, the highest difference in red emission appears during 5 and 7 dpf. Our analysis showed a significant increase in DPP IV activity with respect to the previous stages. The 5 dpf timepoint showed a robust increase in NIR emission intensity over 3 dpf, and we measured the highest intensity value achieved at 7 dpf. Our data are compatible with an increase in DPP IV activity at 5 dpf larvae with a maximum enzymatic activity at 7 dpf larvae (see Figure 7D).

The inhibited 5 dpf zebrafish shows a lower red NIR intensity than the same stage one without inhibitor (see Figure 7D).

## Conclusions

By conjugating the enzyme-recognizing group (Gly-Pro) to the fluorophore (dicyanomethylene-4*H*-pyran derivative, DCM-NH_2_), we synthesized a DPP IV-sensitive and highly specific fluorescent substrate. When the dipeptide group is released from the probe, the donor–acceptor DCM-NH_2_ system is restored, showing the NIR characteristic ICT emission spectrum, which allows us to obtain a ratiometric fluorescence output between the green fluorescent signal of the substrate and the NIR signal of DCM-NH_2_.

Both the substrate and probe are capable of being excited by two NIR photons, which has made it possible to eliminate the green and red bands of autofluorescence from raw plasma and to propose a unique and novel methodology to analyze the activity of DPP IV in raw plasma from diabetic patients. Moreover, the applicability of this new probe as an intracellular *in vivo* sensor of DPP IV activity, as well as its ability to obtain clear fluorescence microscopy images of tumor tissues when excited by two photons, has been confirmed. In addition, the fluorophore released by enzymatic action has been found to be suitable for superresolution fluorescence microscopy imaging. Finally, zebrafish embryos and larvae at 1, 3, 5, and 7 dpf were incubated with the probe. Zebrafish embryos at 1 dpf show red fluorescence mainly in the yolk sac, while older zebrafish larvae show red fluorescence in the yolk sac and in the central nervous system, eye, throat, and digestive tract. We quantified the NIR intensity values at the different stages, with the oldest fish being the most fluorescent in the red, indicating an increase in DPP IV levels with developmental stages. All of these findings make it promising to apply our probe in other biologically relevant situations where DPP IV is overexpressed, like cancer and diabetes.

## Supporting Information Available

Synthesis of DCM-NH-Pro-Gly; mass spectrum of compound DCM-NH-Pro-Gly; ^1^H NMR spectrum of compound DCM-NH-Pro-Gly; ^13^C NMR spectrum of compound DCM-NH-Pro-Gly; molar extinction coefficient of DCM-NH-Pro-Gly; emission spectra; solvatochromic study; mass spectrum; HPLC-MS; HPLC calibration; emission kinetics at different sitagliptin concentrations; ratiometric measurements at different sitagliptin concentrations; temperature and pH enzymatic activity dependence; Kavanagh law; fluorescence intensities at different substrate concentrations; increase of DCM-NH_2_ at different substrate concentrations; Michaelis–Menten model; Lineweaver–Burk representation; excitation spectra of plasma; emission spectra of plasma; comparison of emission spectra at different DMSO concentrations; fluorescence intensity of plasma/DMSO; emission kinetics of DCM-NH-Pro-Gly with DPP IV in plasma/DMSO; spectra resulted of subtracting blank; emission kinetics of DCM-NH-Pro-Gly in plasma/DMSO; emission kinetics of DCM-NH-Pro-Gly in diabetic plasma/DMSO; ratiometric measurements over time of fluorescence signals; two-photon excitation spectra; intensity values of green and red channels and ratio values using excitation wavelengths of 488 and 800 nm of blood plasma sample with DCM-NH_2_ and DCM-NH-Pro-Gly; MTT assay; images obtained from live Caco-2 cells using confocal and STED microscopy; control living zebrafish embryos and larvae; confocal images of living zebrafish embryos and larvae; confocal of control living zebrafish embryos and larvae; and image analysis (PDF)
