# Supplementary material for: Ratiometric Two-Photon Near-Infrared Probe to Detect DPP IV in Human Plasma, Living Cells, Human Tissues, and Whole Organisms Using Zebrafish
Source: ACS Sens. 2023 Feb 27;8(3):1064–75. doi: 10.1021/acssensors.2c02025 (PMC10043939; doi:10.1021/acssensors.2c02025)
Supplement: Supplementary file 1 — se2c02025_si_001.pdf [file se2c02025_si_001.pdf]

Supporting Information

Ratiometric Two-Photon Near Infrared Probe to Detect DPP IV in Human Plasma, Living Cells, Human Tissues and Whole Organisms using Zebrafish

Javier Valverde-Pozo[1], Jose M. Paredes[1, *], Thomas J. Widmann[2], Carmen Griñan-Lison[2, 3], Giada Ceccarelli [5], Antimo Gioiello[5], M. Eugenia Garcia-Rubiño[1], Juan A. Marchal[4, 6, 7], Jose M. Alvarez-Pez[1], Eva M. Talavera[1,*]

1 Nanoscopy-UGR Laboratory, Department of Physical Chemistry, Faculty of Pharmacy, Unidad de Excelencia en Quimica Aplicada a Biomedicina y Medioambiente (UEQ), University of Granada, C. U. Cartuja, 18071 Granada, Spain.

2 GENYO, Centre for Genomics and Oncological Research, Pfizer/University of Granada/Andalusian Regional Government, 18016 Granada, Spain.

3 Instituto de Investigación Biosanitaria (ibs.GRANADA), 18012 Granada, Spain.

4 UGC de Oncología Médica, Complejo Hospitalario de Jaen, 23007 Jaen, Spain.

5 Laboratory of Medicinal and Advanced Synthetic Chemistry (Lab MASC), Department of Pharmaceutical Sciences, University of Perugia, Via del Liceo 1, 06123, Perugia, Italy

6 Centre for Biomedical Research (CIBM), Biopathology and Regenerative Medicine Institute (IBIMER), University of Granada, 18100 Granada, Spain.

7 Department of Human Anatomy and Embryology, Faculty of Medicine, University of Granada, 18016 Granada, Spain.

**Table of contents**	**Page**
Figure S1. Synthesis of DCM-NH-Pro-Gly	S3
Mass spectrum of compound DCM-NH-Pro-Gly	S4
^{1}H-NMR spectrum of compound DCM-NH-Pro-Gly	S4
^{13}C-NMR spectrum of compound DCM-NH-Pro-Gly	S5
Figure S2. Molar extinction coefficient and emission spectra	S6
Quantum yield calculation and Table S1	S7
Figure S3. Solvatochromic study	S8
Figure S4. Mass spectrum	S9
Figure S5. HPLC-MS	S10
Figure S6. HPLC calibration	S12
Figure S7. Emission kinetics at different sitagliptin concentrations	S13
Figure S8. Ratiometric measurements at different sitagliptin concentrations	S14
Figure S9. Temperature and pH enzymatic activity dependence	S14
Figure S10. Kavanagh law	S15
Figure S11. Fluorescence intensities at different substrate concentrations	S15
Figure S12. Increase of DCM-NH_2 at different substrate concentrations	S16
Figure S13. Michaelis-Menten model	S16
Figure S14. Lineweaver-Burk representation	S17
Figure S15. Excitation spectra of plasma	S17
Figure S16. Emission spectra of plasma	S18
Figure S17. Comparison of emission spectra at different DMSO concentrations	S18
Figure S18. Fluorescence intensity of plasma/DMSO	S19
Figure S19. Emission kinetics of DCM-NH-Pro-Gly with DPP IV in plasma/DMSO	S19
Figure S20. Spectra resulted of subtracting blank	S20
Figure S21. Emission kinetics of DCM-NH-Pro-Gly in plasma/DMSO	S21
Figure S22. Emission kinetics of DCM-NH-Pro-Gly in diabetic plasma/DMSO	S22
Figure S23. Ratiometric measurements over time of fluorescence signals	S23
Figure S24. Two photon excitation spectra	S24
Figure S25. Intensity values of green and red channels and ratio values using excitation wavelengths of 488 and 800 nm of blood plasma sample with DCM-NH_2	S25
Figure S26. Intensity values of green and red channels and ratio values using excitation wavelengths of 488 and 800 nm of blood plasma sample with DCM-NH-Pro-Gly	S25
Figure S27. MTT assay	S26
Figure S28. Images obtained from live Caco-2 and cells using confocal and STED microscopy	S27
Figure S29. Control Living zebrafish embryos and larvae	S28
Figure S30. Confocal images of living zebrafish embryos and larvae	S28
Figure S31. Confocal of control living zebrafish embryos and larvae	S29
Image analysis	S29

Synthesis of DCM-NH-Pro-Gly:

To a solution of compound 1 [1] (60 mg, 0.192 mmol) in anhydrous DMF (1.2 mL) under an Ar atmosphere, HATU (0.292 g, 0.77 mmol) and DIPEA (99.2 mg, 0.77 mmol) were added. The mixture was then cooled to 0 °C and stirred for 10 min. Then, a solution of commercially available N-Boc-Pro-Gly (0.105 g, 0.385 mmol) in anhydrous DMF (1.8 mL) was added dropwise, and the solution was stirred overnight and allowed to reach room temperature. Then, the work-up was made as following: $NaHCO_3$ (30 mL) and CH_2Cl_2 (10 mL x3) were used in the extraction step. The organic layers were combined and then washed with 15 % LiCl solution (30 mL x 2), H_2O (30 mL) and Brine (30 mL), water (25 mL×3) and dried (Na_2SO_4). Finally, the solvent was removed under low pressure, and the residue was submitted to flash chromatography in CH_2Cl_2:CH_3OH + TEA mixtures to produce DCM-Boc-Pro-Gly as a red solid in quantitative recovery.

Compound 2, DCM-Boc-Pro-Gly (89 mg, 0.156 mmol) was dissolved in a 1:74 mixture of CH_2Cl_2/TFA (2.21/0.89 mL), and the resulting mixture was stirred for approximately 5 h at room temperature until no starting material was observed by TLC. Then, the solvent was removed under low pressure, and the residue was submitted to flash chromatography to produce 65 mg (87 %) of compound 3 **DCM-NH-Pro-Gly** as an orange solid. Fusion point: 160-170 °C. **^{1}H-NMR** (600 MHz, DMSO-*d4*) δ 10.29 (s, 1H), 8.73 (d, J = 8.2 Hz, 1H), 7.92 (t, J = 7.6 Hz, 1H), 7.79 (d, J = 8.4 Hz, 1H), 7.75 – 7.70 (m, 5H), 7.61 (t, J = 7.6 Hz, 1H), 7.41 (d, J = 15.8 Hz, 1H), 6.99 (s, 1H), 4.47 (s, 1H), 3.49 (s, 2H), 2.11 – 1.74 (m, 6H), 1.21 (s, 2H). **^{13}C-NMR** (150 MHz, DMSO-*d4*) δ 158.95 (C), 153.40 (C), 152.51 (C), 141.57 (C), 138.76 (CH), 135.77 (CH), 129.42 (2-CH), 126.64 (CH), 125.26 (CH), 119.74 (2-CH), 119.54 (CH), 118.41 (CH), 117.75 (C), 117.59 (C), 116.40 (C), 106.85 (CH), 61.36 (C), 60.74 (CH), 60.23 (C), 47.18 (C), 46.12 (CH_2), 39.97 (C), 29.81 (CH_2), 29.48 (CH_2), 24.84 (CH_2). **HRMS (ESI)** *m/z* $[M]^+$ calculated for $C_{27}H_{23}N_5O_3$ 466.1879; obtained: 466.1902.

Fig. S1. Synthesis of compound DCM-NH-Pro-Gly

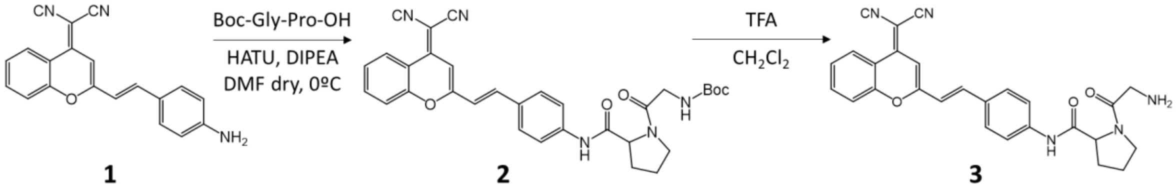

Mass spectrum of compound DCM-NH-Pro-Gly

Elemental Composition Report **Page 1**

Single Mass Analysis
Tolerance = 10.0 PPM / DBE: min = -1.5, max = 50.0
Element prediction: Off
Number of isotope peaks used for i-FIT = 3

Monoisotopic Mass, Even Electron Ions
34 formula(e) evaluated with 1 results within limits (up to 50 best isotopic matches for each mass)
Elements Used:
C: 0-27 H: 0-1000 N: 0-5 O: 0-3
Muestra Perugia Javier 13 (0.304) AM (Top,1, Ht,5000.0,0.00,1.00)
1: TOF MS ES+

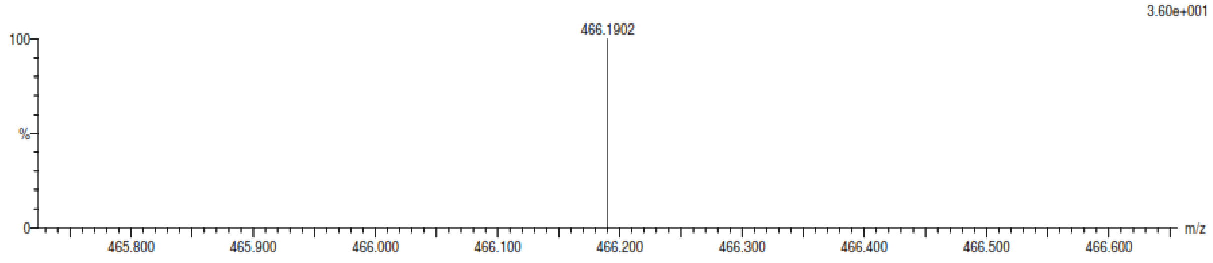

Mass	Calc. Mass	mDa	PPM	DBE	i-FIT	i-FIT (Norm)	Formula
Minimum:				-1.5			
Maximum:		5.0	10.0	50.0			
466.1902	466.1879	2.3	4.9	18.5	16.7	0.0	C27 H24 N5 O3

^{1}H-NMR spectrum of compound DCM-NH-Pro-Gly

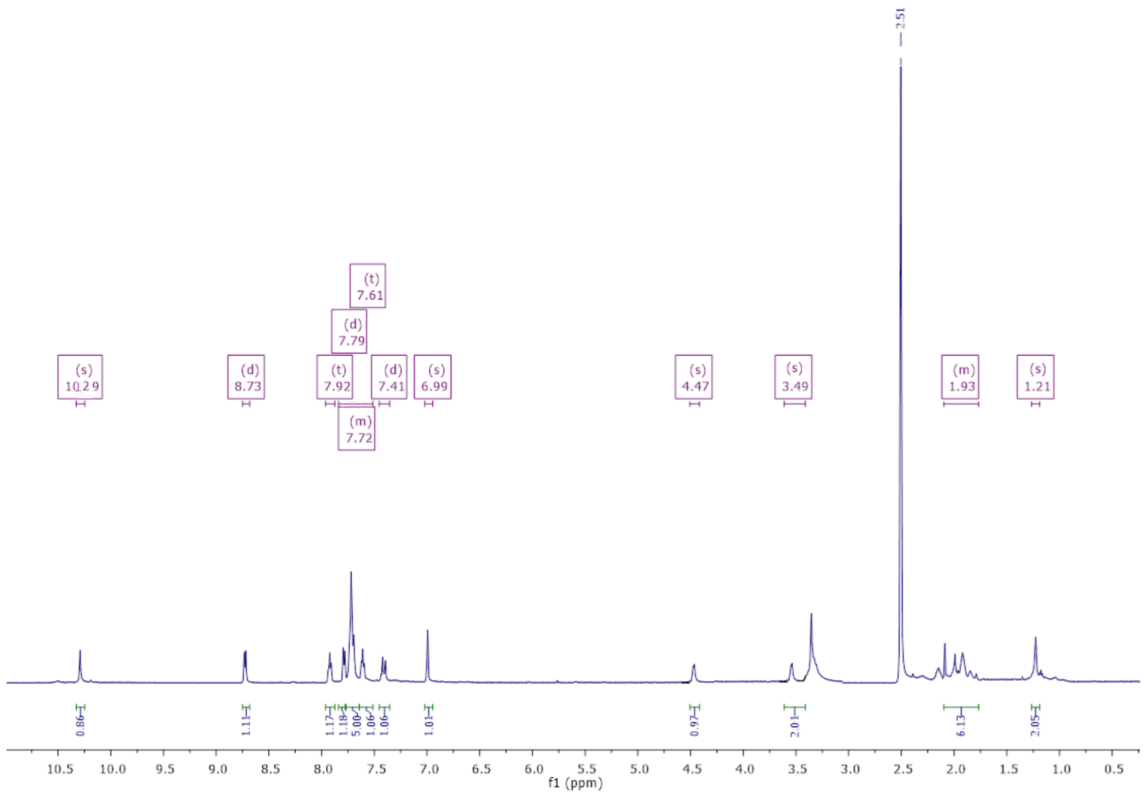

^{13}C-NMR spectrum of compound DCM-NH-Pro-Gly

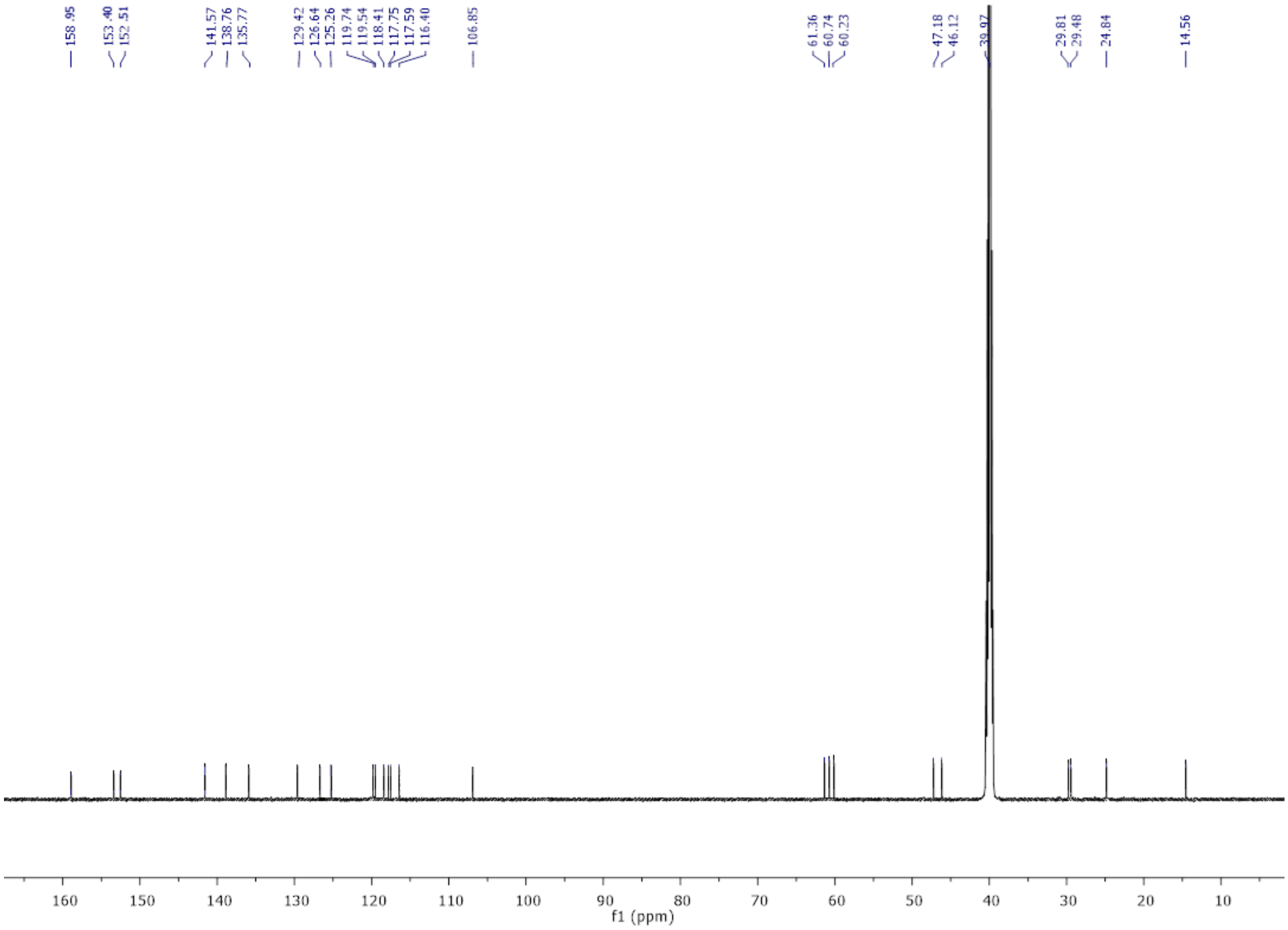

Fig. S2. A) Molar extinction coefficient calculation of DCM-NH-Pro-Gly (ε=17 600 ± 300 L mol^{-1} cm^{-1}; R^2=0.998 and DCM-NH_2 (ε=37 700 ± 1 600 L mol^{-1} cm^{-1}; R^2=0.991). B) Emission spectra of the pure compounds DCM-NH-Pro-Gly (red line) and DCM-NH_2 (black line), 10 μM, in PBS/DMSO 7/3 v/v by excitation at 440 and 480 nm, respectively.

A

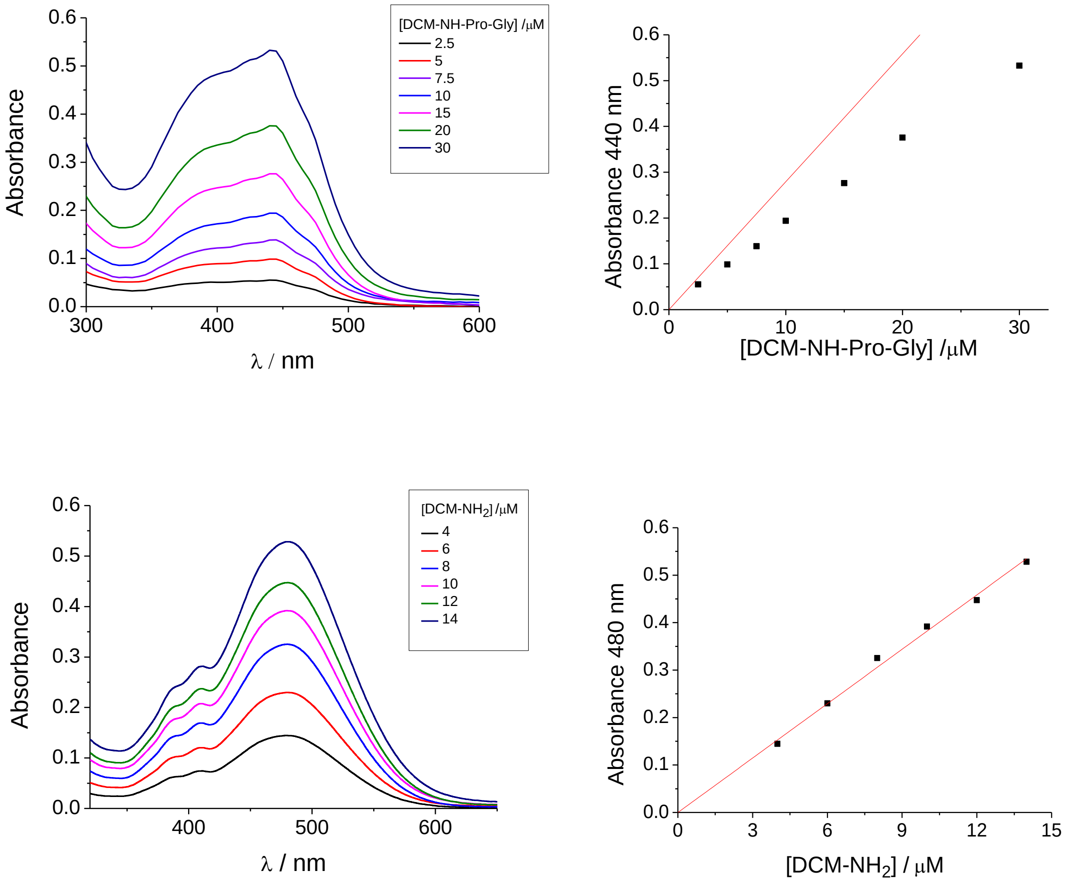

B

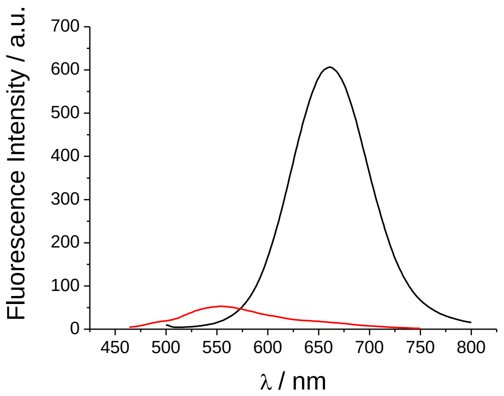

Quantum yield calculation

The relative fluorescence quantum yields have been acquired by integrating the areas under the fluorescence curves using the expression:

$$\Phi = \Phi_R \cdot \frac{I}{I_R} \cdot \frac{OD_R}{OD} \cdot \frac{n^2}{n_R^2}$$

where Φ and Φ_R represent the fluorescence quantum yields of the sample and reference, respectively; I and I_R the integrated fluorescence spectra of the sample and reference, respectively; OD and OD_R are the absorption at the excitation wavelength of the sample and reference, respectively; and n and n_R are the refractive indexes of the solvents in which the sample and reference are dissolved, respectively. As references, we have used Fluorescein in NaOH 0.1M (Φ = 0.91) for DCM-NH-Pro-Gly, and Rhodamine 6G in EtOH (Φ = 0.94) for DCM-NH_2.

Table S1. Quantum yields.

Compound	Solvent	Quantum yield
DCM-NH-Pro-Gly	PBS/DMSO, 7/3, v/v	0.10 ± 0.01%
DCM-NH_2	PBS/DMSO, 7/3, v/v	0.63 ± 0.09%
DCM-NH_2	DMSO	5.73 ± 0.09%

Fig. S3. A) Absorption and B) emission spectra of the compound DCM-NH_2, 1.7 μM in different solvents.

A

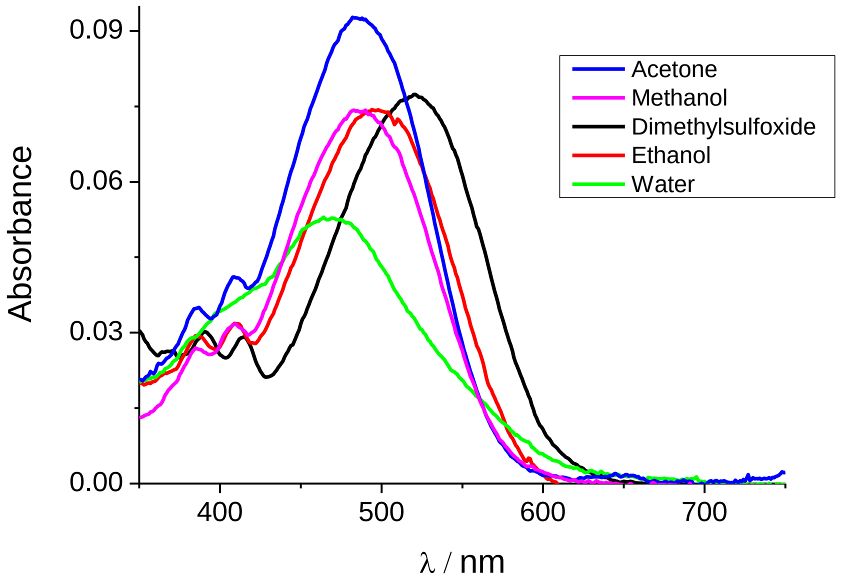

B

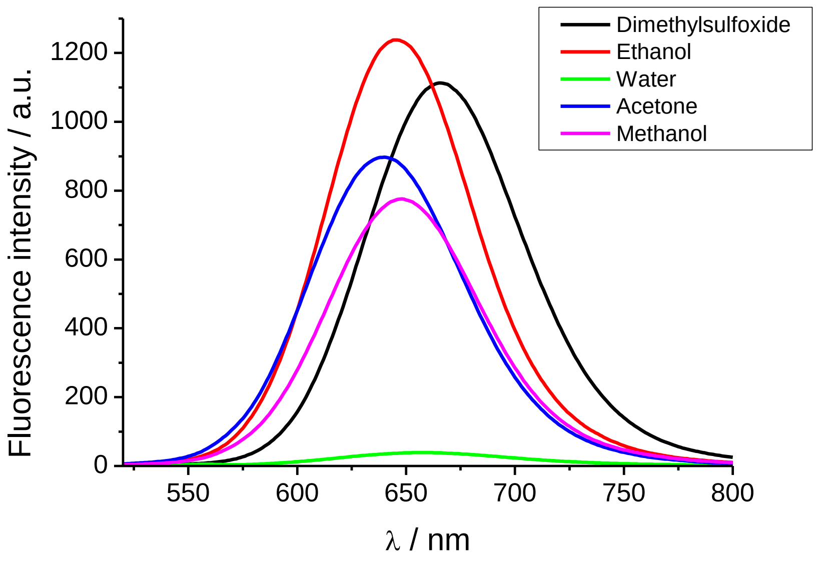

Fig. S4. Mass spectrometry of the compound DCM-NH_2

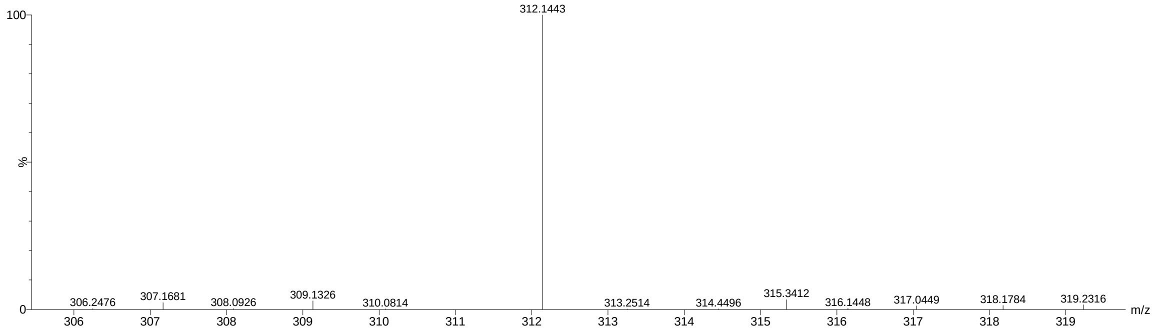

Fig. S5. HPLC-MS of the probe. The arrow indicates the M^+ = 312.14 at different incubation times with DPP-IV A) initial time B) 30 min C) 60 min and D) 90 min.

A

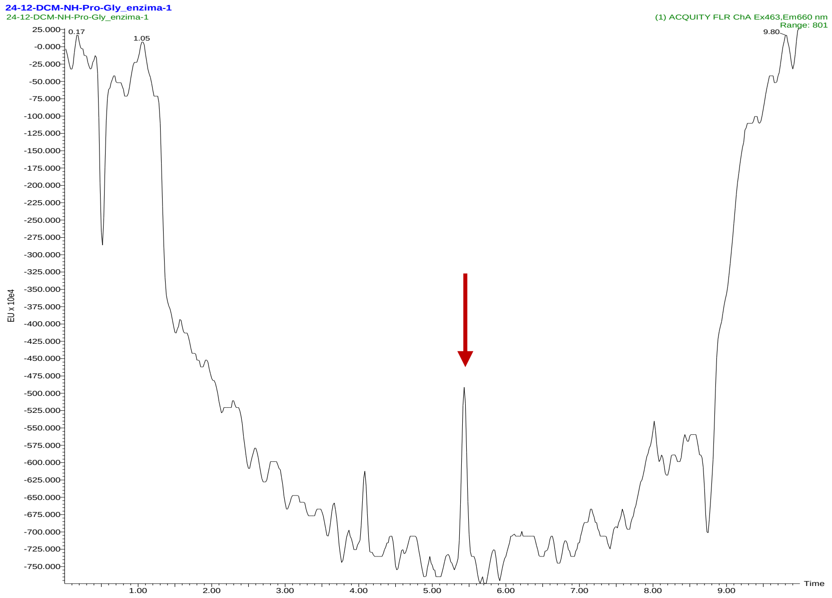

B

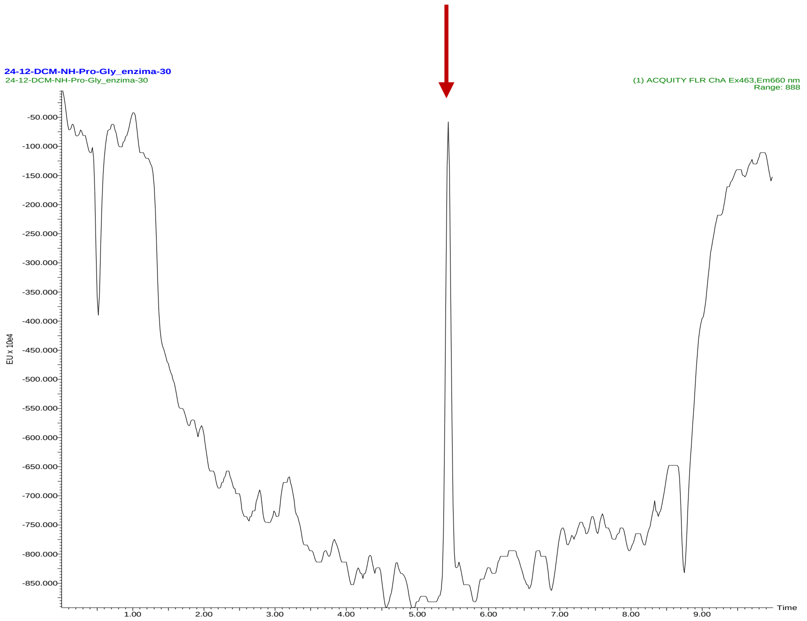

C

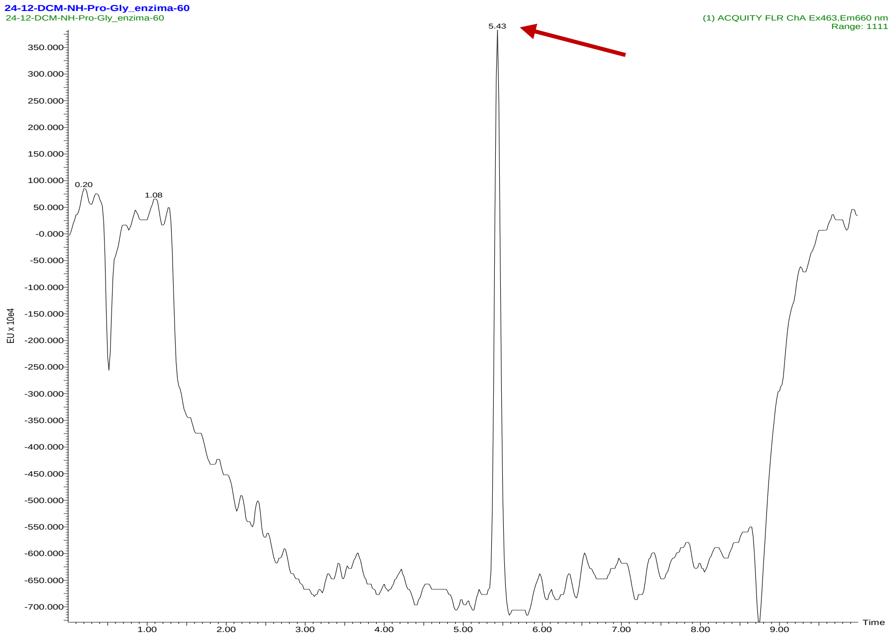

24-12-DCM-NH-Pro-Gly_enzima-60
24-12-DCM-NH-Pro-Gly_enzima-60
(1) ACQUITY FLR ChA Ex463,Em660 nm
Range: 1111
5.43
0.20
1.08
EU x 10e4
Time

D

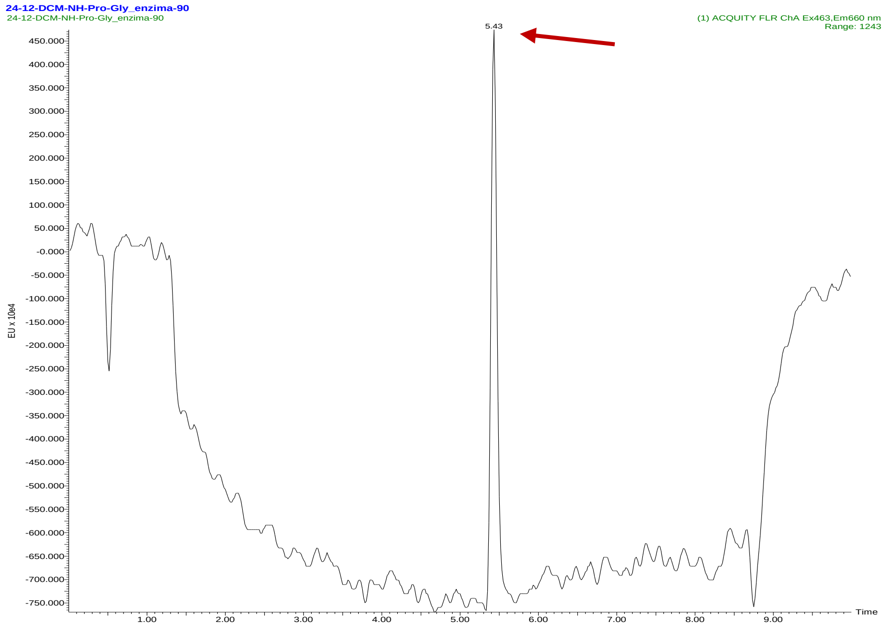

24-12-DCM-NH-Pro-Gly_enzima-90
24-12-DCM-NH-Pro-Gly_enzima-90
(1) ACQUITY FLR ChA Ex463,Em660 nm
Range: 1243
5.43
EU x 10e4
Time

Fig. S6. A) Calibration of the HPLC-MS with differente concentration of DCM-NH_2. B) DCM-NH_2 released at different incubation times with DPP-IV using a [DCM-Pro-Gly] = 1 μM and [DPP-IV] = 5 μg mL^{-1} in PBS /DMSO, 7/3 v/v.

A

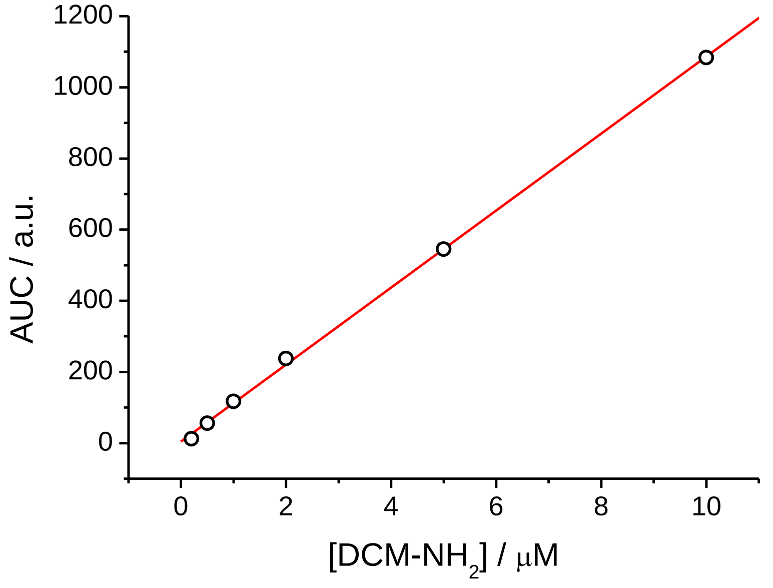

B

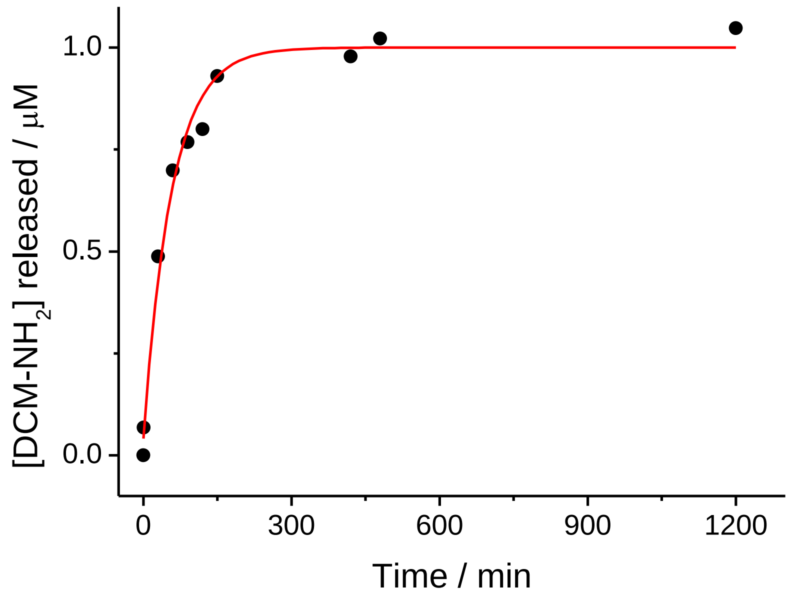

Fig. S7. Emission kinetics of DCM−NH-Pro-Gly (10 μM) with DPP IV (5 μg mL^{-1}) at different sitagliptin concentrations for 2h by excitation at 463 nm at 37 °C.

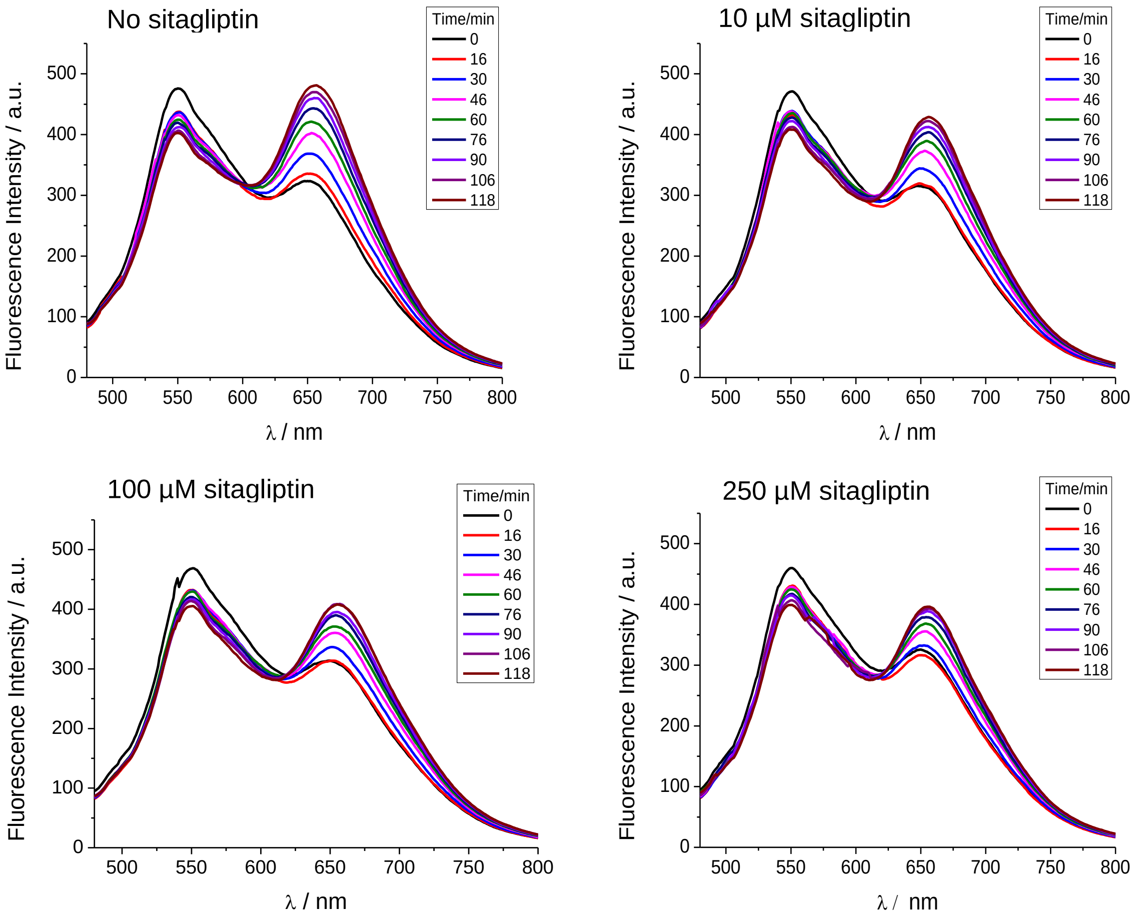

Fig. S8. Ratiometric measurements of fluorescence signals of I_{662} / I_{550} of DCM-NH-Pro-Gly over time with different concentrations of sitagliptin.

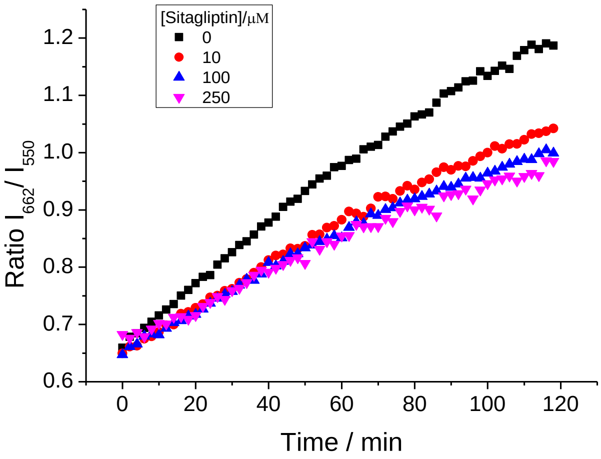

Fig. S9. A) Emission spectra of DCM−NH−Pro-Gly (10 μM) with DPP IV (5 μg mL^{-1}) after 60 min of incubation at 37 ºC and different pHs by excitation at 463 nm. Ratiometric measurements of fluorescence signals of I_{662}/I_{550} of DCM-NH-Pro-Gly (10 μM) with DPP IV (5 μg mL^{-1}) after 60 min of incubation at 37 ºC and different pHs by excitation at 463 nm. B) Emission spectra of DCM−NH−Pro-Gly (10 μM) with DPP IV (5 μg mL^{-1}) after 60 min of incubation at different temperatures and pH 7.5 by excitation at 463 nm. Ratiometric measurements of fluorescence signals of I_{662}/I_{550} of DCM-NH-Pro-Gly (10 μM) with DPP IV (5 μg mL^{-1}) after 60 min of incubation at different temperatures and pH 7.5 by excitation at 463 nm.

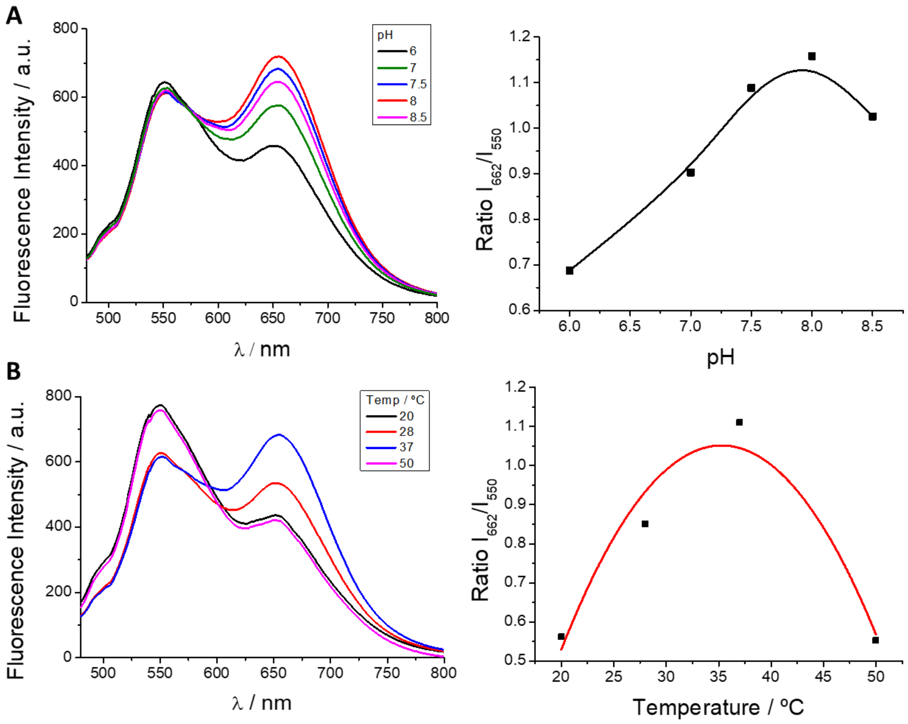

Fig. S10. Fluorescence intensities at 662 nm of DCM-NH_2 at different concentrations by excitation at 550 nm. From the linear fit, we have obtained a slope of 1 707 ± 27 a.u. μM^{-1} and an intercept of 129 ± 33 a.u. (R^2 =0.996).

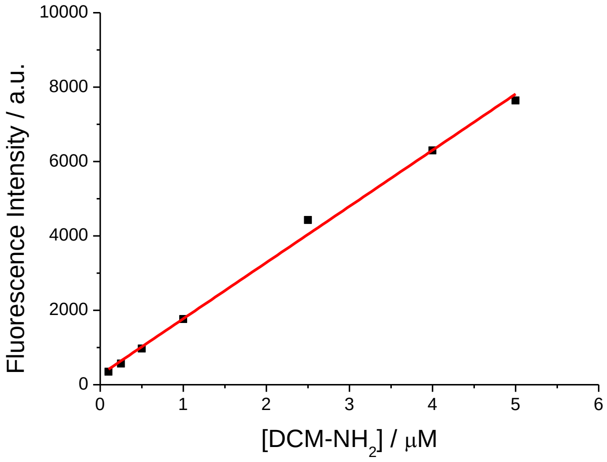

Fig. S11. Fluorescence intensities at 662 nm of DCM-NH-Pro-Gly at different concentrations (1 μM - 15 μM) with DPP IV (2.5 μg mL^{-1}) over time by excitation at 550 nm.

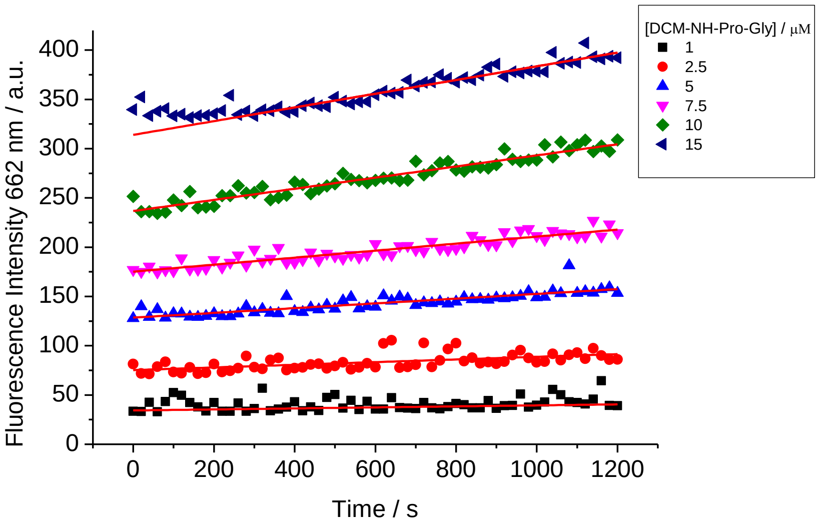

Fig. S12. Increase of DCM-NH_2 concentration (the product of the enzymatic reaction) over time, at different concentrations of DCM-NH-Pro-Gly (1.00 μM – 15.00 μM) with DPP IV (2.5 μg mL^{-1}).

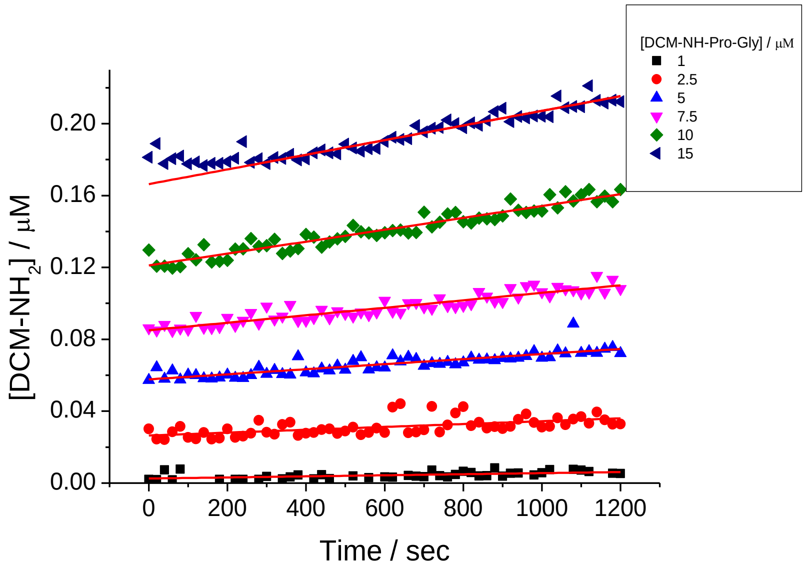

Fig. S13. Initial rates of fluorescence measurements vs substrate concentrations of DCM-NH-Pro-Gly with DPP IV (2.5 μg mL^{-1}).

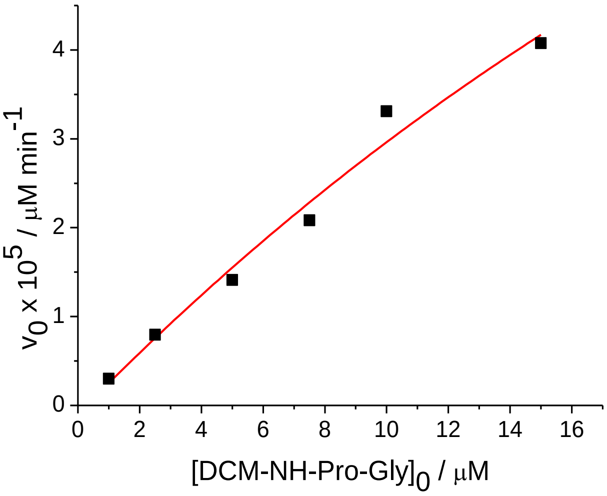

Fig. S14. Lineweaver-Burk representation of the enzymatic reaction between DPP IV and the substrate DCM-NH-Pro-Gly. The linear fit provides a slope of 331 000 ± 6 300 min and an intercept of 680 ± 50 min μM^{-1}, (R^2 =0.998).

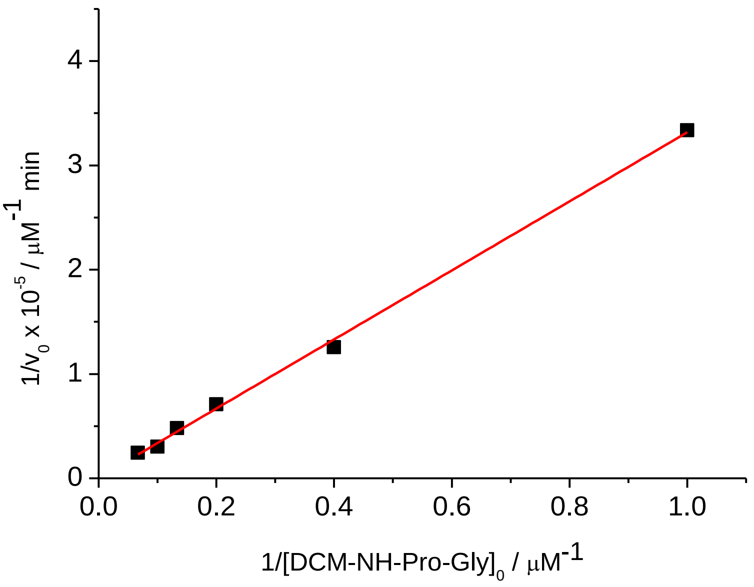

Fig. S15. Excitation spectrum of plasma with emission at 580 nm.

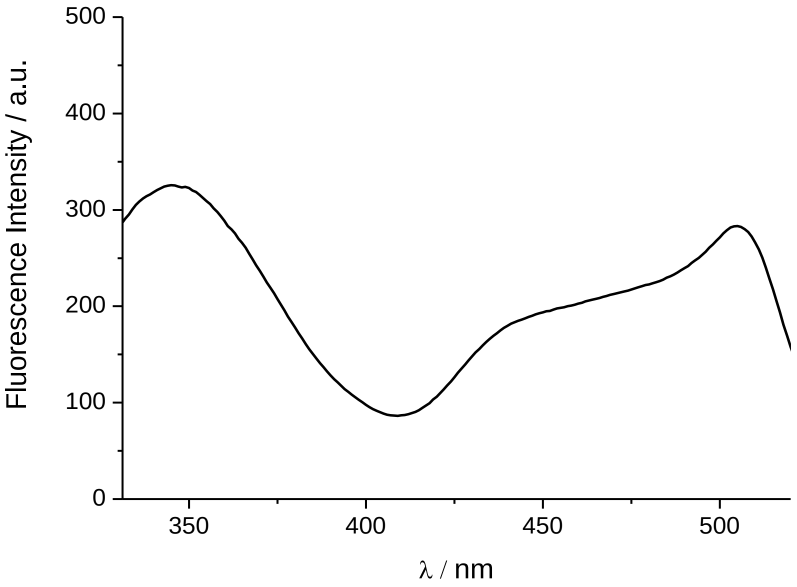

Fig. S16. Emission spectrum of the plasma by excitation at 480 nm.

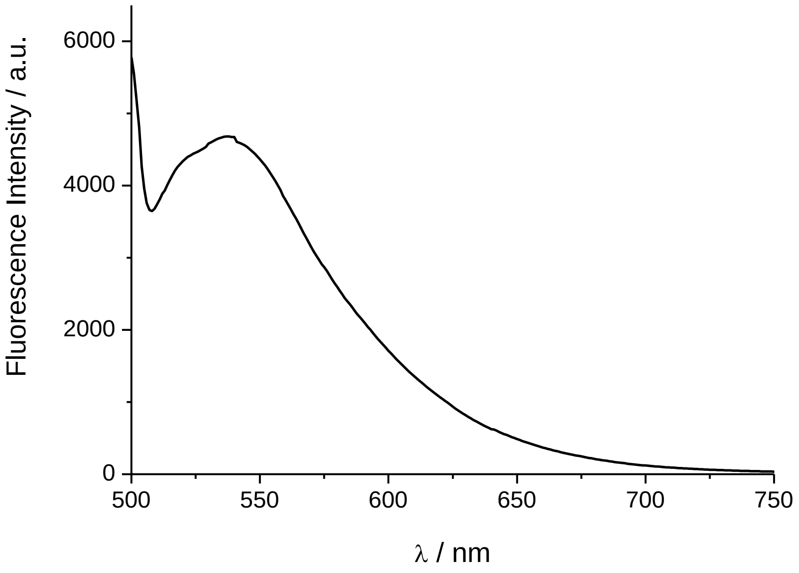

Fig. S17. Comparison of emission spectra of the kinetics of the enzymatic reaction in plasma at different times (0, 24 and 48 h, and 48 h with different concentrations of DMSO, by excitation at 480 nm.

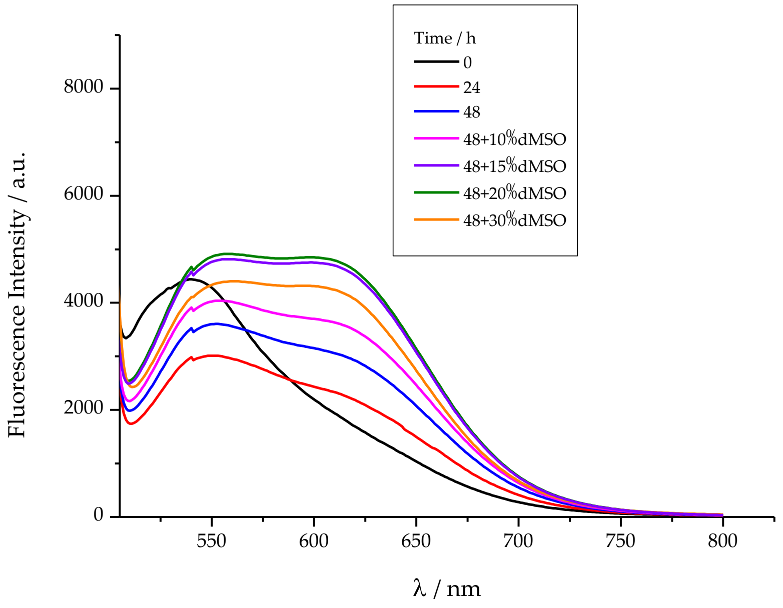

Fig. S18. Fluorescence intensity of plasma/DMSO 85/15, v/v, for 24 h, every hour, by excitation at 480 nm.

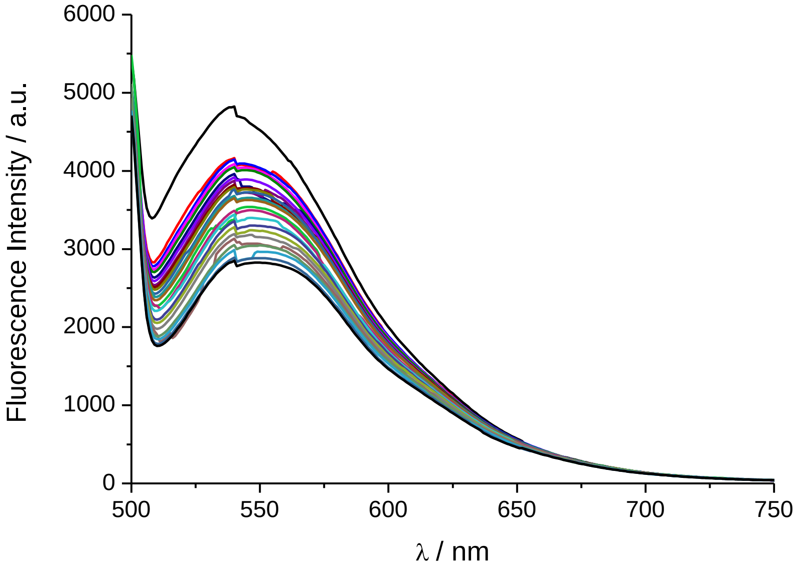

Fig. S19. Emission spectra of kinetics of DCM-NH-Pro-Gly 10 μM with DPP IV 10 μg mL^{-1} in plasma/DMSO 85/15, v/v, for 24 h, every hour, by excitation at 480 nm.

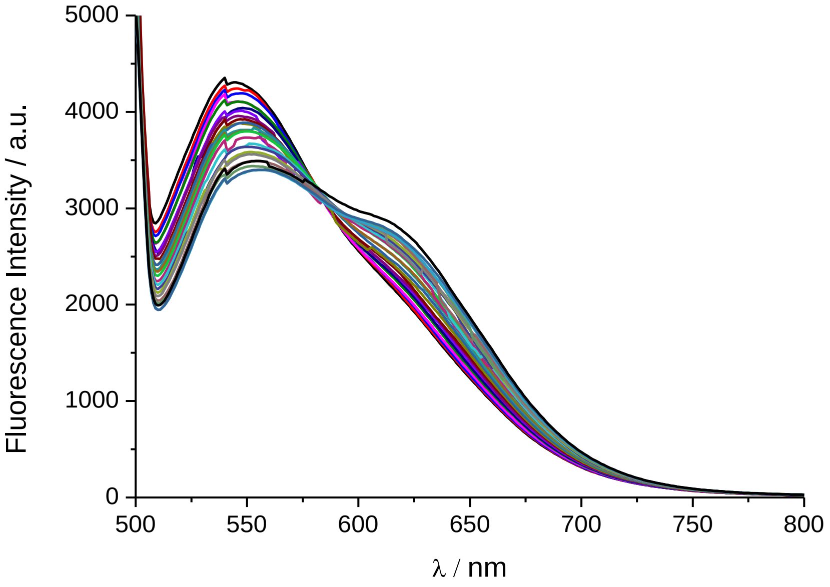

Fig. S20. Result of subtracting from the spectra of Figure S19, the spectra of Figure S18. The Figure includes only the results every 4 h for clearer visualization.

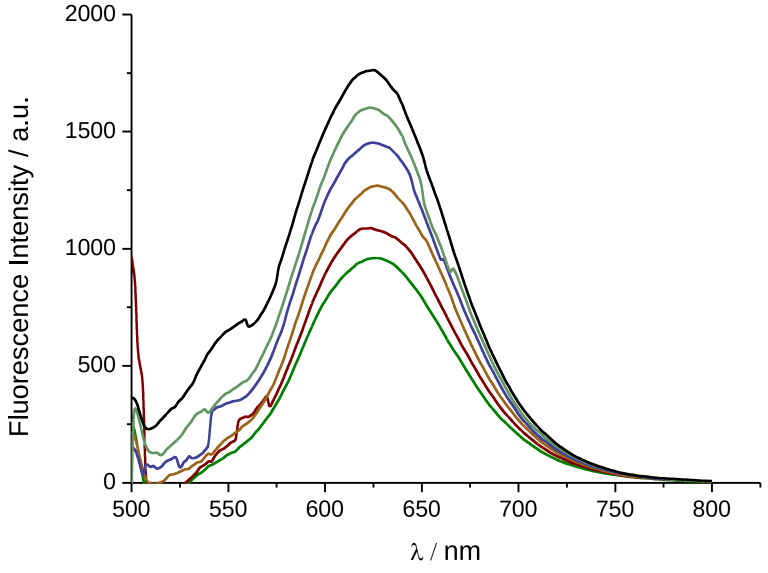

Fig. S21. A) Emission spectra of kinetics of DCM-NH-Pro-Gly 10 µM in plasma/DMSO 85/15, v/v, for 24 h, every hour, by excitation at 480 nm. B) Intensity values at 540 and 635 nm over time.

A

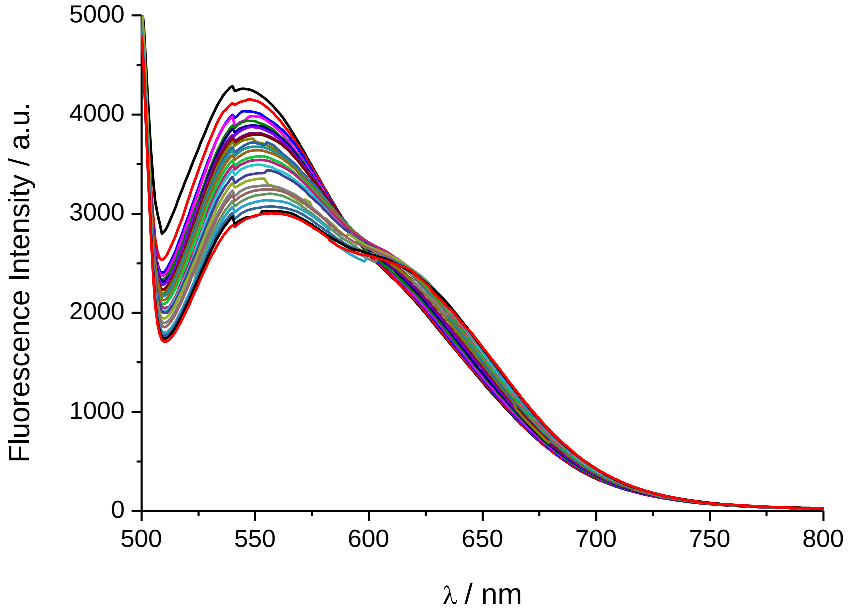

B

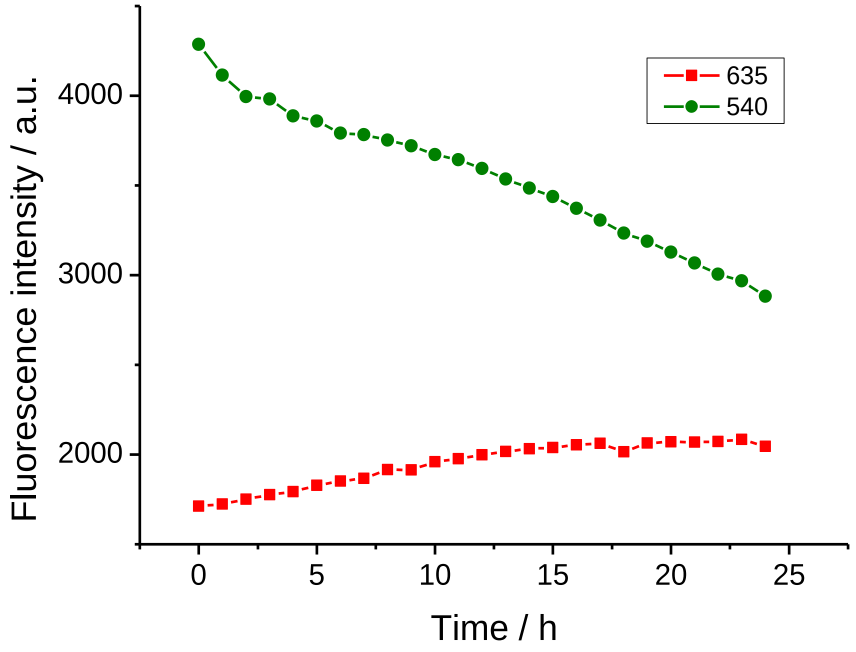

Fig. S22. A) Emission spectra kinetics of DCM-NH-Pro-Gly 10 µM in diabetic plasma/DMSO 85/15, v/v, for 24 h, every hour, by excitation at 480 nm. B) Intensity values at 540 and 635 nm over time.

A

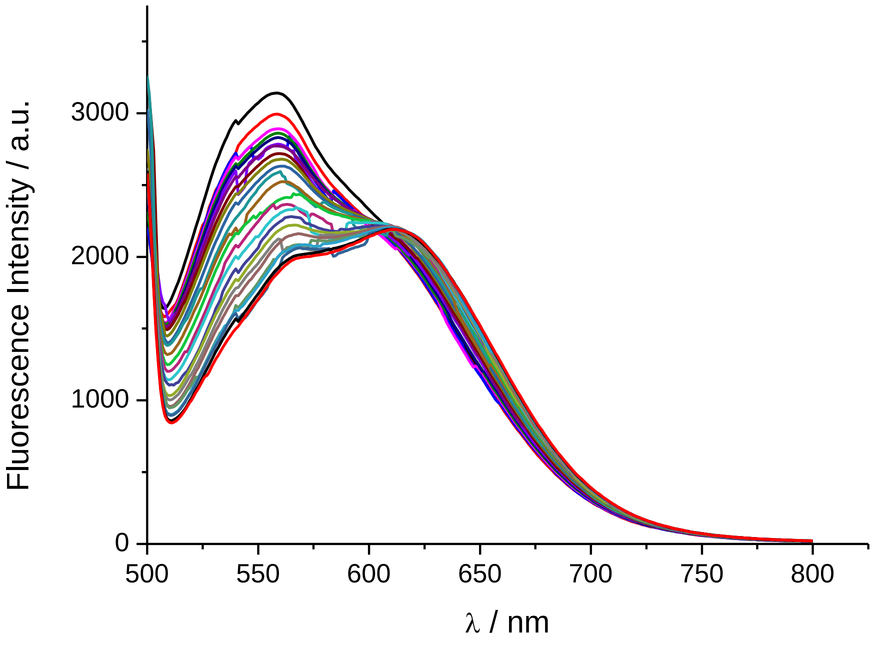

B

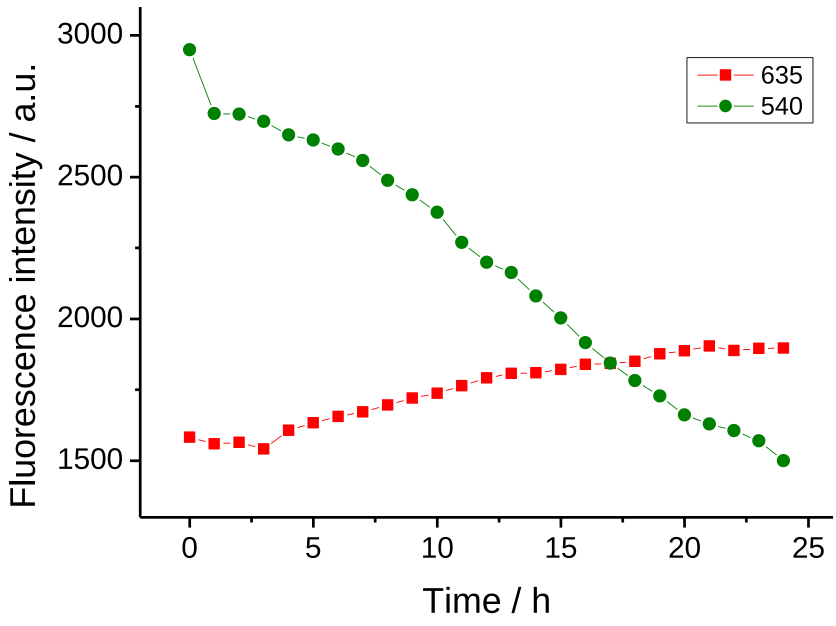

Fig. S23. Ratiometric measurements normalized over time of fluorescence signals of I_{635} / I_{540} of DCM-NH-Pro-Gly in plasmas from subjects diabetic and healthy.

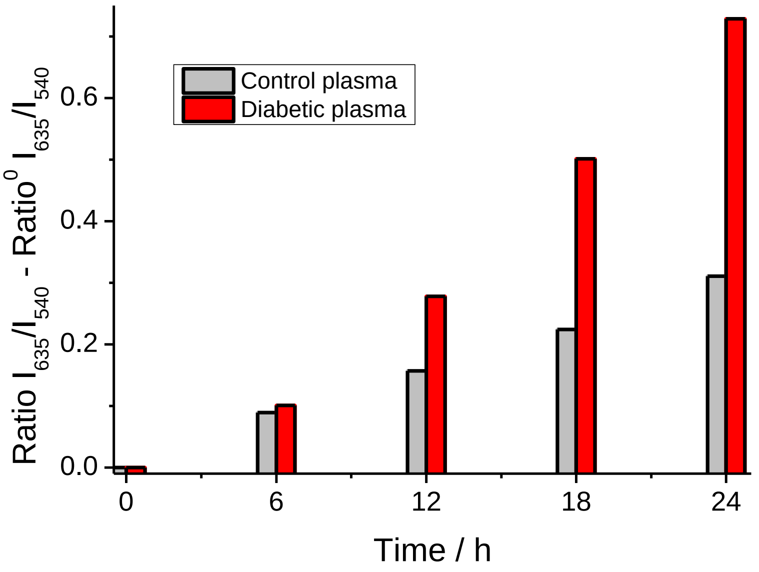

Figure S24. Two-photon excitation spectra of DCM-NH_2 (square in red) and DCM-NH-Pro-Gly (circles in blue) measured in PBS/DMSO 7/3 v/v. Emission collected at 650−720 nm and 502-538 nm, and excitation ranging from 720 to 1000 nm, measured every 20 nm. The intensities were calculated by normalization to the power of the excitation source measured on the excitation pathway. Bars represent the standard error from three replicates.

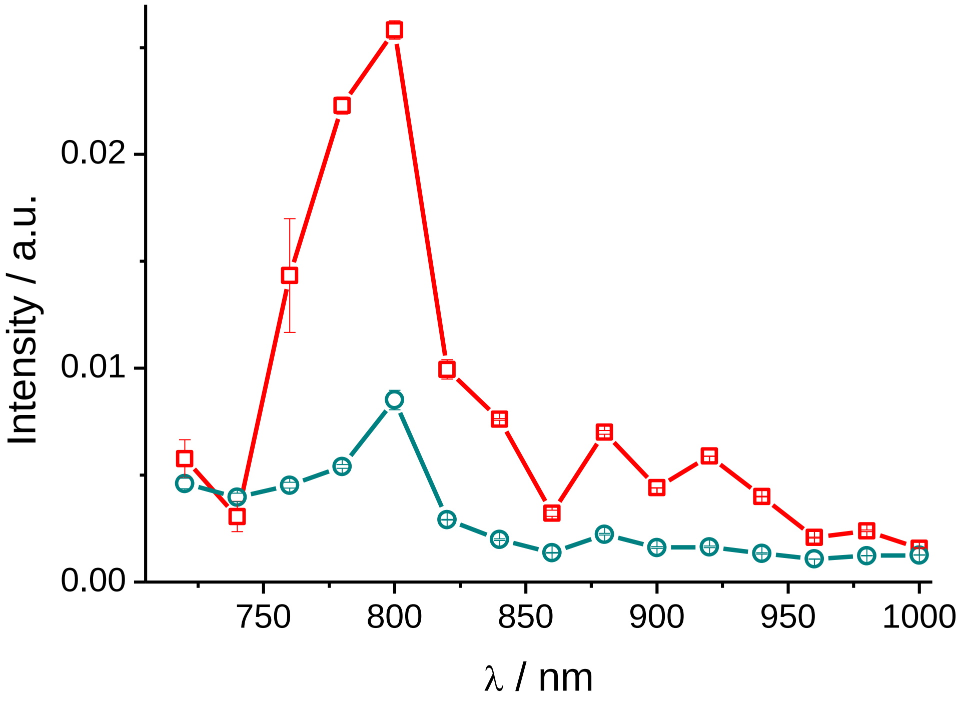

Fig. S25. Intensity values of green and red channels and Ratio maps using excitation wavelengths of 488 and 800 nm of blood plasma sample with DCM-NH_2. Whiskers represent the SE.

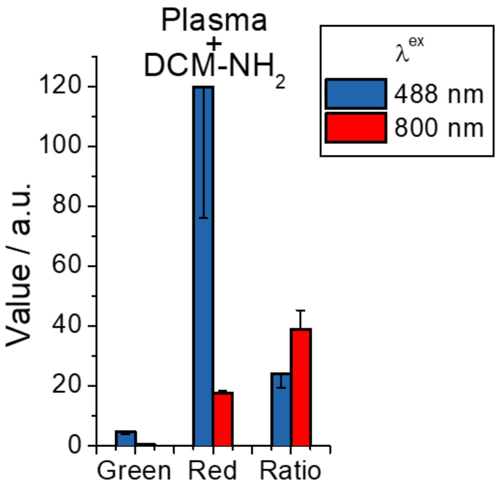

Fig. S26. Intensity values of green and red channels and Ratio maps using excitation wavelengths of 488 and 800 nm of blood plasma sample with DCM-NH-Pro-Gly. Whiskers represent the SE.

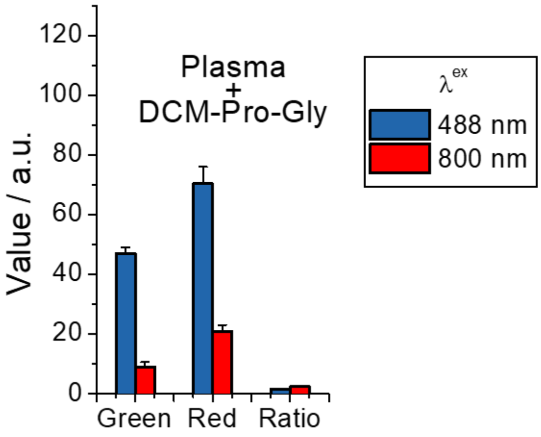

Fig. S27. Proliferation assay (A: Caco-2 cells; B: BxPC-3 cells). Histogram graphics over time using different concentrations of DCM-NH-Pro-Gly (2.5, 5 and 10 μM, corresponding to a percentage of DMSO equal to 0.5, 1 and 2 %, respectively) and compared with DMSO at the same percentages (0.5, 1, and 2 %) and with a control. Data are represented as absorbance at 570 nm and shown as mean ± SE. The statistically significant values are represented with $^{**}p < 0.01$; $^{*}p < 0.05$ (comparing DMSO vs control), $^{\#}p < 0.05$ (comparing DCM-NH-Pro-Gly vs control), $^{\$}p < 0.05$ (comparing DMSO vs DCM-NH-Pro-Gly), by the T-test.

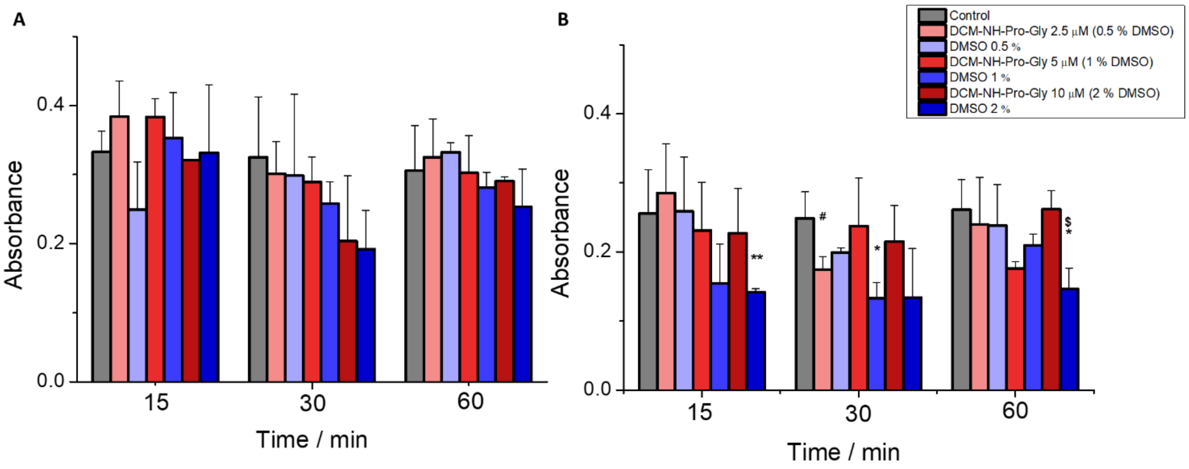

Fig. S28. Images obtained from live Caco-2 cells incubated with DCM-NH_2 (5 μM) using confocal and STED microscopy. The image in the left is a general overview of the cells. The square indicates the zone where the confocal/STED was measured. In the middle we represent the images using confocal/STED microscopy. In the right we represent the plot profile (black corresponds to confocal images and red to STED images) for the line shown in the middle images. Scale bars in the overview image represent 5 µm and in the confocal/STED image represent 0.5 µm.

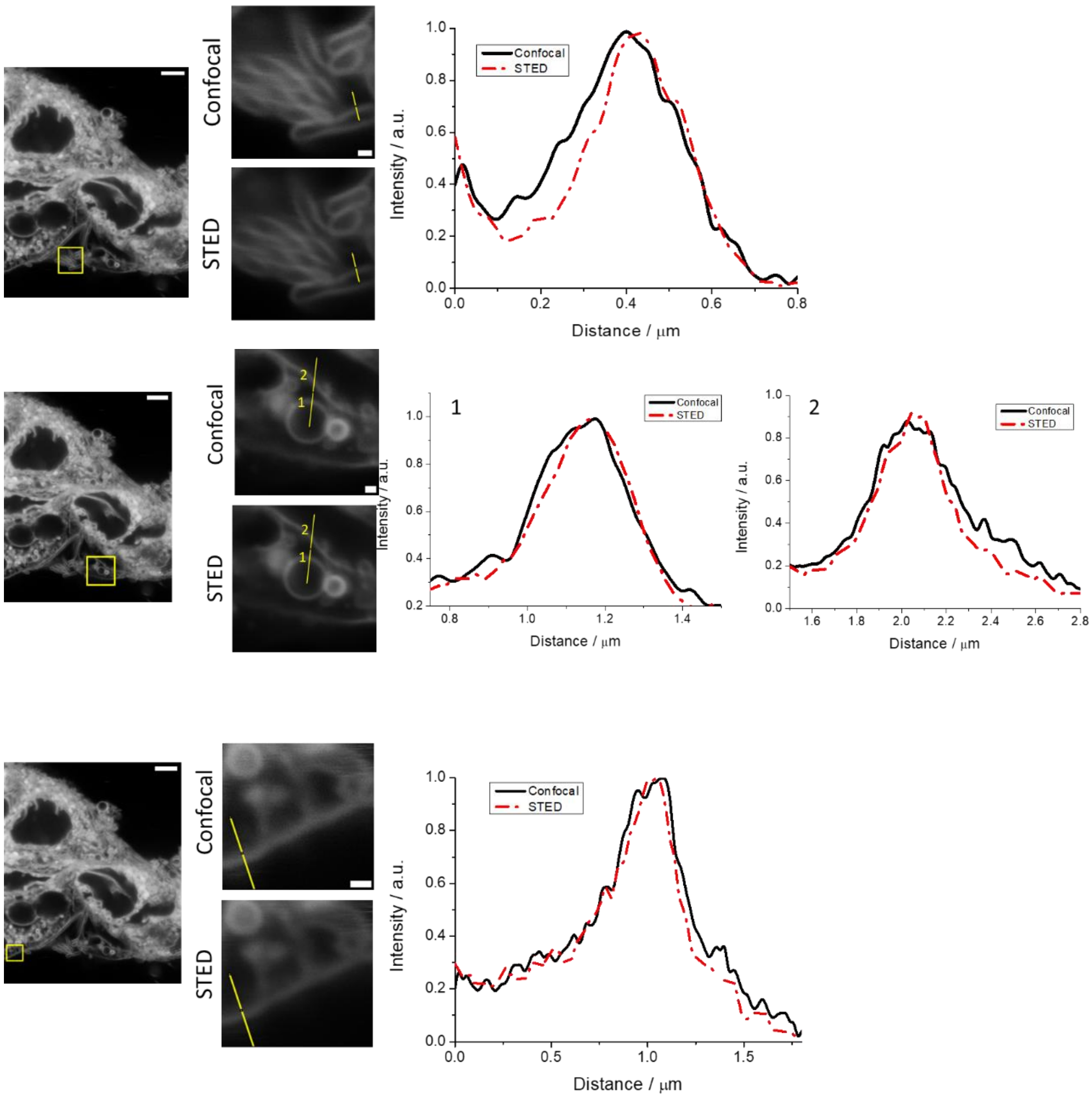

Fig. S29. Living zebrafish embryos and larvae incubated with 5 µM DMSO for 2 h at different days post fertilization (dpf); red fluorescent (left), brightfield (centre), and merge (right) images are taken with a stereo microscope (λ_{exc} = 458 nm; λ_{em} = 680 nm) Scale bars: 1 dpf: 250 µm, 3-7 dpf: 500 µm.

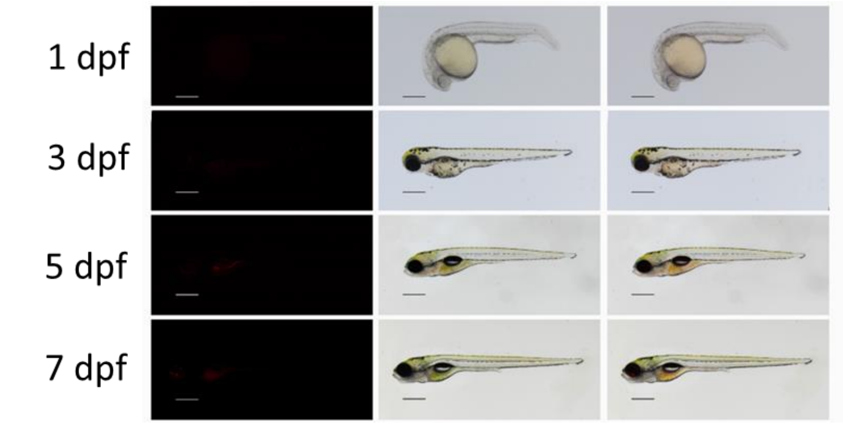

Fig. S30. Living zebrafish embryos and larvae incubated with 5 µM DCM-NH-Pro-Gly for 2 h at different dpf; red fluorescent (left), brightfield (centre), and merge (right) images by confocal microscopy. Each image is composed from different fields (tiles) that were mounted together using the plugin "Pairwise stitching" tool in *Fiji Is Just ImageJ*. For the red channel, maximum projections of z-stacks are shown. CNS refers to central nervous system. Scale bars: 1 dpf: 250 µm, 3-7 dpf: 500 µm, detail 3 dpf: 200 µm.

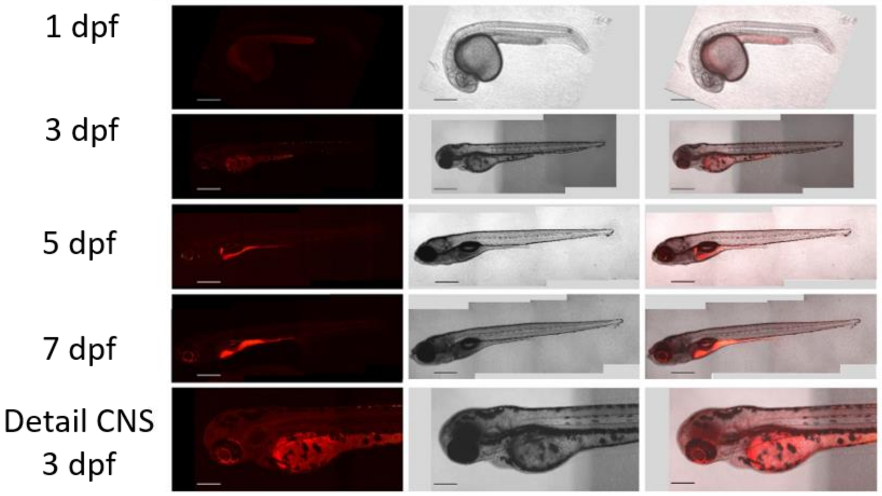

Fig. S31. Living zebrafish embryos and larvae incubated with 5 µM DMSO for 2 h at different dpf; red fluorescent (left), brightfield (centre), and merge (right) images by confocal microscopy. Each image is composed from different fields (tiles) thatwere mounted together using the plugin "Pairwise stitching" tool in *Fiji Is Just ImageJ*. For the red channel, maximum projections of z-stacks are shown. CNS refers to central nervous system. Scale bars: 1 dpf: 250 µm, 3-7 dpf: 500 µm, detail 3 dpf: 200 µm.

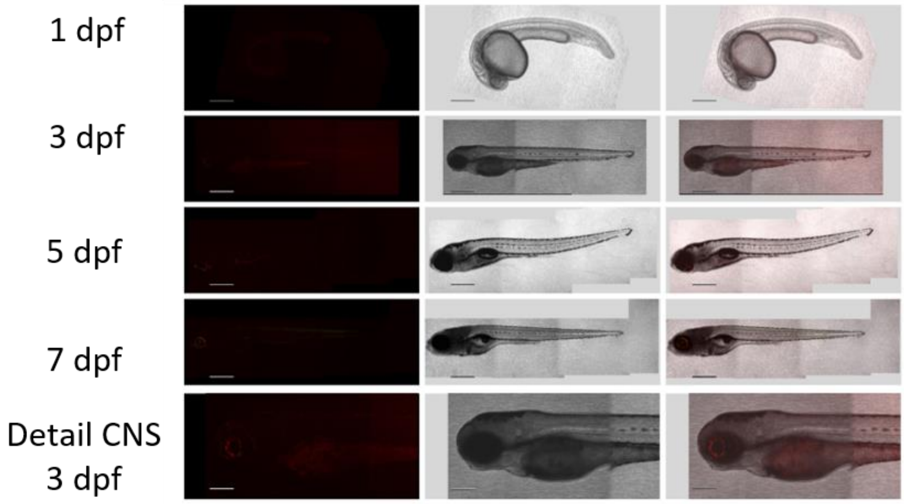

Image analysis.
Image quantification and ratio measurements were performed using home-coded macros in *Fiji Is Just ImageJ*. In brief, a Gaussian filter was performed to the raw images data of cells, tissues and zebrafish (sigma=2) and plasma (sigma=3). For the cells, tissues and zebrafish, a manual threshold selection was performed to select the regions of interest (ROI). The plasma images were used completely. Outside the ROI a Not a Number (NaN) was assigned to pixels. The segmented images were used to calculate the average intensity value in every channel. We obtained the ratio images dividing both segmented channels (red and green) pixel to pixel. The calculated images were used to calculate the average ratio value.
